# Biotransformation of Cholesterol and 16*α*,17*α*-Epoxypregnenolone and Isolation of Hydroxylase in* Burkholderia cepacia* SE-1

**DOI:** 10.1155/2016/5727631

**Published:** 2016-06-02

**Authors:** XiangDong Zhu, CuiPing Pang, Yuting Cao, Dan Fan

**Affiliations:** College of Bioscience and Bioengineering, Jiangxi Agricultural University, Nanchang 330045, China

## Abstract

The metabolism of cholesterol is critical in eukaryotes as a precursor for vitamins, steroid hormones, and bile acids. Some steroid compounds can be transformed into precursors of steroid medicine by some microorganisms. In this study, the biotransformation products of cholesterol and 16*α*,17*α*-epoxypregnenolone produced by* Burkholderia cepacia* SE-1 were investigated, and a correlative enzyme, hydroxylase, was also studied. The biotransformation products, 7*β*-hydroxycholesterol, 7-oxocholesterol, and 20-droxyl-16*α*,17*α*-epoxypregn-1,4-dien-3-one, were purified by silica gel and Sephadex LH-20 column chromatography and identified by nuclear magnetic resonance and mass spectroscopy. The hydroxylase was isolated from the bacterium and the partial sequences of the hydroxylase, which belong to the catalases/peroxidase family, were analyzed using MS/MS analyses. The enzyme showed activity toward cholesterol and had a specific activity of 37.2 U/mg of protein at 30°C and pH 7.0.

## 1. Introduction

Cholesterol has an important role in chemistry, medicine, and biology and is an essential structural component of animal cell membranes [1]. The metabolic products of cholesterol are the precursors of vitamins, steroid hormones, and bile acids [2, 3]. Since Murray and Peterson first discovered that* Rhizopus nigricans* could transform progesterone into 11*α*-hydroxyl progesterone, it has attracted considerable attention [4]. Low-cost natural steroids, such as cholesterol, can be used as starting materials for synthesizing many bioactive steroids [5]. Therefore, an understanding of the bacterial metabolism of cholesterol could be useful in the development of biotechnological tools for the transformation of steroid components via metabolic engineering. Many different microorganisms can use environmental cholesterol and related sterols as a common carbon source. The microbial transformation of steroids has great advantages over chemical processing methods, especially because it is very difficult to process those steroids using chemical methods. Recently, various mechanisms and metabolic pathways for steroids in microbes have been studied. Some bacteria and fungi can hydroxylate at different locations on the steroid molecule [6–8]. Such reactions could be developed into an important technique; particularly, the ability to perform 7-hydroxylation of steroid compounds has potential use. For example,* Acremonium stricture* transformed 4-pregnene-3,20-dione into 7*β*,15*α*-dihydroxyl-4-pregnene-3,20-dione [9]. The filamentous fungus,* Aspergillus fumigatus*, efficiently hydroxylated exogenous progesterone, producing 11*α*- and 15*β*-hydroxyprogesterone as major products and 7*β*-hydroxyprogesterone as a minor product [10]. Bacillus strains transformed 3*β*-hydroxyl-4-pregnene-20-one into 3*β*,7*α*-dihydroxyl-4-pregnene-20-one [11]. Some 7-hydroxyl derivatives of steroid play an important role in medicine. For example, 7-hydroxyl derivatives of dehydroepiandrosterone have a curative effect against cancer and Alzheimer's disease and show antiglucocorticoidal action and decrease fat and enhance immune function [12, 13]. The same functions are exhibited by 7-hydroxyl derivatives of 4-pregnene-3,20-dione [12]. Some studies indicate that 7*β*-hydroxycholesterol can inhibit cell hyperplasia and tumor cell multiplication [14, 15]. In our study, we report for the first time the transformation of cholesterol and 16*α*,17*α*-epoxypregnenolone by strain* Burkholderia cepacia* SE-1. The transformation products, 7*β*-hydroxycholesterol, 7-oxocholesterol, and 20-droxyl-16*α*,17*α*-epoxypregn-1,4-dien-3-one, and a correlative enzyme, hydroxylase, were investigated.

## 2. Materials and Methods

### 2.1. Materials

Cholesterol and 16*α*,17*α*-epoxypregnenolone were purchased from Xian Blue Sky Biological Engineering Co., Ltd. (China). Silica gel GF254 plates and silica gel 200–300 mesh were obtained from Qingdao Marine Chemical Ltd. (China). NMR spectra were recorded on Bruker AV-600 MHz instruments (Bruker, Switzerland) with TMS as an internal standard. MS spectra were recorded on an HPLC-MS ZQ4000/2695 (Waters, USA). Sephadex LH-20 (Amersham Biosciences, Sweden) was used for column chromatography. Petroleum ether, acetone, and chloroform were of analytical grade and methanol for High Performance Liquid Chromatography (HPLC) was of chromatography grade (Waters Symmetry Shield RP 18, 150 mm × 3.9 mm 5 *μ*m).

### 2.2. Methodology

#### 2.2.1. Screening and Isolation of the Bacterium

Strains that metabolized cholesterol were isolated from forest field soil on the campus of Jiangxi Agricultural University, Nanchang, China. Microorganisms that could metabolize cholesterol were screened with a screening medium. The enrichment media were composed of (g/L) Peptone (Aobox, Beijing) 10, Beef Extract (Aobox, Beijing, China) 3, MgSO_4_ 0.25, K_2_HPO_4_ 0.25, FeSO_4_·7H_2_O 0.001, NaCl 0.05, and CaCl_2_ 0.001. The screen media were composed of (g/L) MgSO_4_ 0.25, K_2_HPO_4_ 0.25, FeSO_4_·7H_2_O 0.001, NaCl 0.05, CaCl_2_ 0.001, agar15, pH 7.0, and 1.0 g/L cholesterol as sole carbon source. Cholesterol and sodium dodecyl sulfate (SDS) were mixed together at the molar ratio of 1 : 1 for 20 min with an ultrasonic mixer to increase the solubility of cholesterol in water. The medium was solidified with 1.5% (wt. vol^−1^) agar. The culture medium contained (g/L) Peptone (Aobox, Beijing) 10, Beef Extract (Aobox, Beijing) 3, MgSO_4_ 0.25, K_2_HPO_4_ 0.25, FeSO_4_·7H_2_O 0.001, NaCl 0.05, and CaCl_2_ 0.001 and the microorganisms were grown at 30°C, with shaking at 220 r/min for 24 h. Uninoculated culture medium incubated under the same conditions was used as a control.

#### 2.2.2. Identification of the Bacterium

Only one bacterial species with the desired metabolic activity was isolated and it was identified and characterized using standard biochemical tests from* Bergey's Manual of Determinative Bacteriology* [17]. Cellular morphology was observed with a transmission electron microscope (H7650, Japan). This species was identified by comparing the sequence of the 16S rDNA gene, which was isolated with a UNIQ-10 column genomic DNA kit, to the database of NCBI GenBank, genomic DNA. The DNA was sequenced by a commercial automated sequencing system provided by Sangong Biotech Ltd. (Shanghai, China). The bacterium genomic 16S rDNA was amplified by PCR. The sense (5′-AGAGTTTGATCCTG GTCAG-3′) and antisense (5′-AAGGAGGTGATCCAGCCGCA-3′) primers universal to prokaryotes were used. A phylogenetic tree was constructed using the neighbor-joining method with MEGA (5.0) software. The strain was deposited in the microbial laboratory of the College of Bioscience and Bioengineering, Jiangxi Agricultural University.

#### 2.2.3. Assay of Biotransformation Products

A single colony of* Burkholderia cepacia* SE-1 from an agar plate of the screening medium was inoculated into 30 mL of culture medium in 100 mL flasks and incubated at 30°C, while shaking at 220 rpm for 24 h. The culture was subsequently inoculated into 300 mL of culture medium in 1000 mL flasks and incubated at 30°C and 220 rpm for 24 h. Cells were collected by centrifugation at 8000 ×g for 10 minutes at room temperature and then resuspended in 300 mL of reaction mixture containing 0.07 M Na_2_HPO_4_/KH_2_PO_4_ buffer (pH 8.0) and 1.0 g/L cholesterol or 16*α*,17*α*-epoxypregnenolone in a 1000 mL flask and incubated at 30°C and 220 rpm for 48 h. After the reaction was complete, cells were harvested from the culture by centrifugation for 10 minutes at 8000 ×g, at room temperature. Cells were sequentially extracted with 50, 30, and 20 mL chloroform, respectively. Therefore some products were solved into chloroform. The chloroform solution was collected and evaporated* in vacuo*. The chloroform solution (5 *μ*L) of transformed products was spotted onto a 0.25 mm silica gel GF254 thin layer chromatography (TLC) plate, developed in a petroleum ether : acetone (3 : 1, v/v) solvent system and visualized by heat after spraying with 10% H_2_SO_4_-anhydrous ethanol.

#### 2.2.4. Purification and Structural Characterization of the Biotransformation Products

The concentrating chloroform solution was mixed with a little silica gel and subjected to silica gel column chromatography (CC, silica gel (200–300 mesh)) and eluted with petroleum ether : acetone (95 : 5–70 : 30) by monitoring with TLC. The fraction containing the conversion products was purified by Sephadex LH-20 gel column chromatography methods and eluted with CHCl_3_-MeOH 1 : 1. 7*β*-Hydroxycholesterol was tested by HPLC. The column was eluted with methanol at a flow rate of 1.0 mL/min. The elution was monitored by measurement of the absorbance at 215 nm. ESI-MS spectra of the purified compounds were acquired on an HPLC-MS ZQ4000/2695 quadrupole LC-MS in the positive and negative modes. NMR spectra were recorded in CDCl_3_-d on a Bruker AM-600 NMR instrument (600 MHz for ^1^H NMR and 125 MHz for ^13^C NMR).

#### 2.2.5. Hydroxylase Activity Was Assayed by TLC

To hydroxylase activity assay, 0.1 mL of a cholesterol solution (10 mg cholesterol dissolved in 1 mL of acetone) was added to 0.5 mL of the elute, 0.2 mM NAD^+^ was added as a cofactor, and the mixture was incubated at 30°C, pH 7.0 for 1 h. Petroleum ether (0.5 mL) was added to the reaction mixture. The solution was shaken and allowed to settle and then 5 *μ*L of the petroleum ether solution was spotted onto a 0.25 mm silica gel GF_254_ TLC plate, developed in petroleum ether : acetone (3 : 1, v/v) solvent system and visualized by heat after spraying with 10% H_2_SO_4_-anhydrous ethanol.

#### 2.2.6. Purification of the Enzyme

*Burkholderia cepacia* SE-1 was grown at 30°C for 24 h in the culture medium and then the culture was centrifuged at 4,000 ×g for 10 minutes at 4°C. Cells were dissolved in a buffer solution of 10 mM Tris-HCl, pH 8.0, containing 0.1 mM EDTA, lysed ultrasonically, and centrifuged at 8,000 ×g for 30 minutes at 4°C. The supernatant was extracted with an (NH_4_)_2_SO_4_ solution at 70% saturation at 4°C for 24 hours. The precipitate was recovered by centrifugation at 12,000 ×g for 30 minutes at 4°C and redissolved in a buffer solution containing 10 mM Tris-HCl, pH 8.0. This solution was dialyzed against the same buffer at 4°C until a 10 mL solution was achieved. The solution was placed on a column (2.5 × 50 cm) of DEAE-cellulose preequilibrated with 10 mM Tris-HCl, pH 8.0, buffer and washed with a linear gradient of Tris-HCl buffer solutions containing 40–120 mM NaCl, at a flow rate of 60 mL/h. The elute (20 mL) was collected in a conical flask.

The fraction of activity of cholesterol hydroxylase was going to be separated further. The fractions were freeze-dried to a 5 mL volume and loaded on a Sephadex G-200 (Pharmacia, Uppsala, Sweden) column (2.5 × 50 cm). This column had been preequilibrated with a 10 mM Tris-HCl (pH 8.0) buffer containing 50 mM NaCl and was eluted with the same buffer at a flow rate of 30 mL/h. The elute (10 mL) was collected in conical flasks. The hydroxylase activity was assayed by TLC as described above. The fractions containing cholesterol hydroxylase were dialyzed against 10 mM Tris-HCl buffer (pH 8.0) and freeze-dried to a 0.5 mL volume and then subjected to polyacrylamide gel electrophoresis. After electrophoresis, the protein bands were cut from two sides of the gel and stained with 0.025% (w/v) of Coomassie Brilliant Blue R-250 in 40% (v/v) methanol and then destained in 50% methanol. By comparing the other section of the gel which was unstained with the two pieces of gel section stained, the protein bands of interest were located and excised and dissolved in 10 mM Tris-HC. The activity in the protein bands was assayed by TLC as described above. All of the purification steps were performed at 4°C. Protein was detected by absorbance at 280 nm.

#### 2.2.7. The Hydroxylase Activity Was Evaluated by HPLC

The hydroxylase activity was assayed by measuring the quantity of 7*β*-hydroxycholesterol produced. Petroleum ether was used to extract 7*β*-hydroxycholesterol from the reaction mixture, which was solubilized with methanol after petroleum volatilization.

One unit of enzymatic activity was defined as the amount of protein required to hydroxylate 1 *μ*mol of cholesterol per minute at 30°C. Reverse-phase chromatography on an ODS column (HPLC) was employed to determine 7*β*-hydroxycholesterol with a preparation of 7*β*-hydroxycholesterol (98%) as the standard. The column was eluted with methanol at a flow rate of 1.0 mL/min and the elution was monitored by measurement of the absorbance at 205 nm.

#### 2.2.8. Amino Acid Sequence Analysis

Purified enzyme was sent to Sangong Biotech Ltd. (Shanghai, China) for MS and MS/MS analyses with matrix assisted-laser desorption ionization- (MALDI-) time of flight (TOF) and MALDI-TOF-TOF methods.

Protein was digested with trypsin and then analyzed with a 4700 Proteomics Analyzer (Applied Biosystems, Foster City, CA, USA). The MALDI MS parameters were designed with MS acquisition in the reflector mode, positive ion mode, mass range 850–4,000 (mass/charge (*m/z*)), and minimum signal/noise (S/N) set at 10 for MS acquisition. Twenty-five of the strongest precursors were chosen for MS/MS, and a minimum S/N 30 was used for MS/MS precursors. The Applied Biosystems GPS Explorer*™* v3.6 program was used for a combined search of MS and MS/MS data, with Mascot as the search engine (available at http://www.matrixscience.com/). Searches allowed for carbamidomethylation and monoisotopic oxidation, 100 ppm of peptide mass or parent tolerance, and a maximum of one missed trypsin cleavage. Peptide tolerance and MS/MS tolerance were both 0.5 Da. Proteins with a statistically significant (*P* < 0.05) protein score were considered as identified with confidence (based on combined mass and mass/mass spectra). Redundancy of proteins that appeared in the database under different names and accession numbers was eliminated.

## 3. Results

### 3.1. Identification of the Microorganism

**Figure 1:**
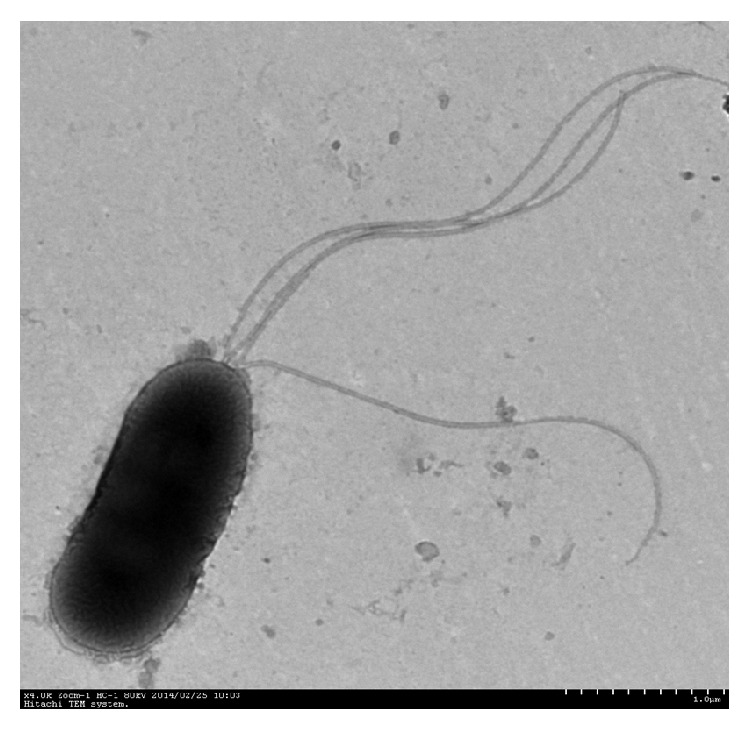
Scanning electron micrograph of* Burkholderia cepacia* SE-1 strain, ×32K.

The isolated organism was rod-shaped and 0.8–1.0 *μ*m × 1.6–3.2 *μ*m in size, Gram-negative, with a bipolar flagellum. The bacterial colony was round, smooth, moist, and opaque, with smooth edges, and had a straw yellow color after 24 h growth at 36°C on the plate. An electron micrograph showing strain morphology is shown in Figure 1.

**Figure 2:**
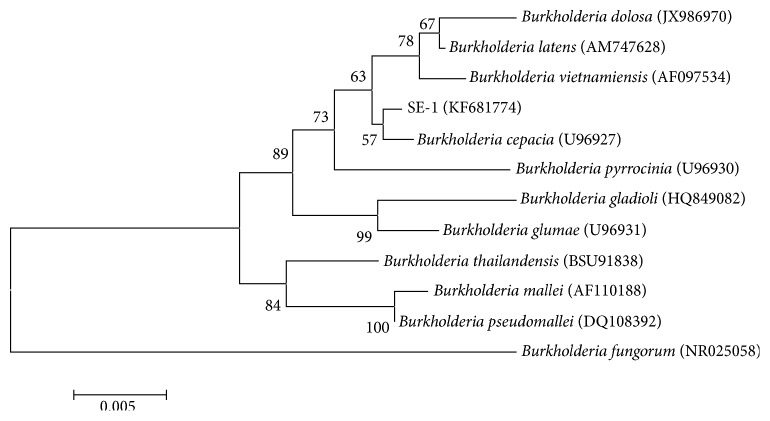
Phylogenetic tree based on 16S rRNA sequences of selected strains. Numbers in parentheses represent the sequences accession number in GenBank. The number at each branch point is the percentage supported by bootstrap. Bar, 0.5% sequence divergence.

We analyzed a partial sequence of the 16S rDNA sequence of this strain. The 16S rDNA gene (approximately 1525 bp) from the isolated bacterial strain was sequenced and the data was submitted to GenBank, which was assigned the accession number KF681774. The amplified sequence showed a 99% similarity to the 16S rRNA genes of* Burkholderia cepacia* strain 17759 from the American Type Culture Collection (ATCC) (GenBank accession number AY741334, results not shown) and* B. cepacia* ATCC 17762 (GenBank accession number AY741335). We built a phylogenetic tree by comparing the sequence of the 16S rDNA from our isolate with sequences from 11 standard* Burkholderia* genus stains. Based on the morphological characteristics and 16S rDNA sequence analysis, our strain was identified as* Burkholderia cepacia *SE-1. The phylogenetic tree based on the 16S rDNA sequence showed that strain SE-1 was classified as* Burkholderia* sp. (Figure 2). No one has yet reported cholesterol hydroxylase from the genus* Burkholderia*, so we studied the cholesterol hydroxylase from the isolated* Burkholderia cepacia *SE-1 strain further.

### 3.2. Separation and Analysis of Biotransformation Products

After the reaction was complete, cells were harvested from the culture by centrifugation for 10 minutes at 8000 ×g, at room temperature. The supernatant and cells were extracted with chloroform, respectively. The thin layer chromatography (TLC) plate showed a deep blue spot in the solution of extracted cells from strain SE-1. It revealed that the blue substance did not exist in the culture medium without inoculated strain and the supernatant. It also proved that hydroxylase enzyme is expressed intracellularly and the biotransformed products are expressed intracellularly. 7*β*-Hydroxycholesterol (128 mg) and 7-oxocholesterol (32 mg) were separated from the reaction mixture with a silica gel and Sephadex LH-20 gel column chromatography. Similarly, 20-droxyl-16*α*,17*α*-epoxypregn-1,4-dien-3-one was separated from the reaction mixture of 16*α*,17*α*-epoxypregnenolone.

### 3.3. Structure Determination of the Biotransformation Products

To characterize the structure of the product, the purified compounds were subjected to MS and NMR analyses. The ion peak [M − H]^+^ at* m/z* 401 and [M + Na]^+^ at* m/z* 425 in the ESI-MS spectra of compound** 1** indicated a formula of C_27_H_46_O_2_. The analysis of the ^13^C spectral data showed 27 carbon signals (Figure S1 in Supplementary Material available online at http://dx.doi.org/10.1155/2016/5727631). A signal of *δ*143.5 and *δ*125.5 with sp^2^ character suggested a double-doublet at C-5 and C-6 in DEPT data (Figure S2). This was affirmed by hydrogen shifts at *δ*5.28 (1H, t, *J* = 2.4 Hz) in the ^1^H spectrum data (Figure S3). The carbon signal of *δ*71.4 (C-3) and 73.4 (C-7) showed that a hydroxyl group linked the A ring at C-3 and another hydroxyl group linked the B ring at C-7. We compared the shift value at *δ*125.5 (C-6) and 40.9 (C-8) of the product with *δ*121.6 (C-6) and 31.9 (C-8); *β*-OH can cause the chemical shift to increase greater than 4.8–7.4 downfield than *α*-OH. Based on this information from MS, ^1^H NMR, and ^13^C NMR, product** 1** was characterized as 7*β*-hydroxycholesterol. This also agreed with the data in the literature [18]. The chemical structures of compound** 1** were seen in Figure 4. The ^1^H and ^13^C NMR spectral data for the product was in Figures S14 and S15.

The analysis of the ESI-MS spectrum data of product** 2** showed the ion peak [M + H]^+^ at* m/z* 401.6. The analysis of the ^13^C spectrum data of it showed 27 carbon signals. The data indicated a formula of C_27_H_44_O_2_ (Figure S4). The signal of *δ*165.0 and *δ*126.2 with sp^2^ character suggested a double-doublet at C-5 and C-6 in DEPT data (Figures S5 and S6). This was affirmed by hydrogen shifts at *δ*5.69 (^1^H, s, H-6) of the ^1^H spectrum data (Figure S7). The carbon signal of *δ*70.6 (C-3) showed that a hydroxyl group linked the A ring at C-3. Based on the information from MS, ^1^H NMR, and ^13^C NMR, compound** 2** was characterized as 7-oxocholesterol. This was in accord with the data in literature [19]. The chemical structures of compound** 2** were seen in Figure 4. The ^1^H and ^13^C NMR spectrum data of compound** 2** was in Figures S14 and S15.

**Figure 3:**
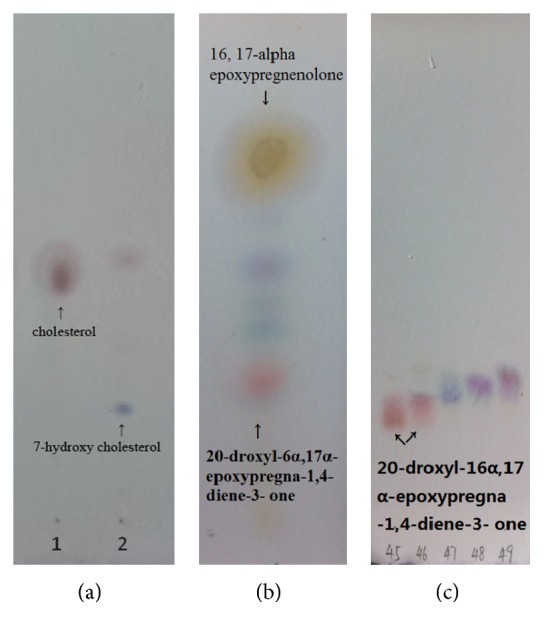
TLC of the reaction solution of the steroids compounds. (a) TLC of the reaction solution of enzyme from strain SE-1 adding cholesterol. (b) TLC of the reaction solution of 16*α*,17*α*-epoxypregnenolone from strain SE-1. (c) 20-Droxyl-16*α*,17*α*-epoxypregn-1,4-dien-3-one being purified.

**Figure 4:**
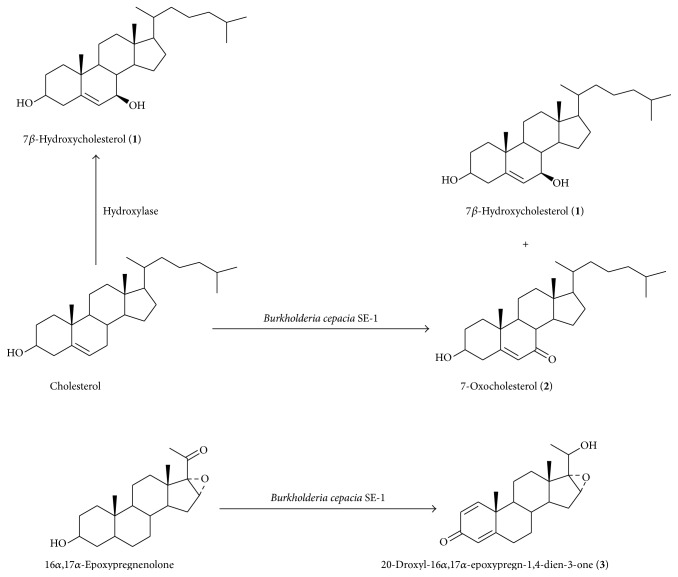
The chemical structures of cholesterol and 16*α*,17*α*-epoxypregnenolone and their hydroxylated substrates.

The analysis of the ESI-MS spectrum data of product** 3** showed the ion peak [M + H]^+^ at* m/z* 329.2 and [M + Na]^+^ at* m/z* 351.2 and [M + K]^+^ at* m/z* 367.1 in the ESI-MS spectra agreed with the formula C_21_H_28_O_3_. The ^13^C spectrum revealed 21 carbon signals (Figure S8): these were sorted by DEPT experiments into CH_3_ × 3, CH_2_ × 5, CH × 8, and C × 5. The signal of *δ*155.1 (d, C-1), 127.7 (d, C-2), 124.1 (d, C-4), and *δ*168.3 with sp^2^ character suggested a double-doublet at C-1 and C-2 and another double-doublet at C-4 and C-5 in DEPT data (Figures S9 and S10). The signal at *δ*186 showed a carbonyl group. These formed a conjugate system. This was affirmed by HSQC and HMBC experiments. The signal at *δ*7.01 (1H, d, *J* = 10.4 Hz), 6.22 (1H, dd, *J* = 10.4, 1.6 Hz), and 6.06 (1H, s) correlated with the *δ*155.1 (d, C-1), 127.7 (d, C-2), and 124.1 (d, C-4), respectively (Figure S11). The signal at *δ*7.01 (1H, d, *J* = 10.4 Hz) correlated with the *δ*168.3 (s, C-5) and 21.1 (q, C-19) and *δ*6.06 (1H, s) correlated with the *δ*127.4 (s, C-2), 33.1 (t, C-6), and 45.2 (s, C-10) in HMBC spectrum (Figure S12). The carbon signal of *δ*69.2 (d, C-20) showed a hydroxyl group at C-20. Based on the information from MS, ^1^H NMR, and ^13^C NMR, compound** 3** was characterized as 20-droxyl-16*α*,17*α*-epoxypregn-1,4-dien-3-one. The TLC plate showed a red spot that indicated that 16*α*,17*α*-epoxypregn-1,4-dien-3-one has been transformed into 20-droxyl-16*α*,17*α*-epoxypregn-1,4-dien-3-one (Figure 3). The chemical structures of compound** 3** were seen in Figure 4. The ^1^H spectrum data of 3 was in Figures S13 and S14. The ^13^C NMR spectrum data of 3 was in Figure S15.

### 3.4. Enzyme Purification

**Figure 5:**
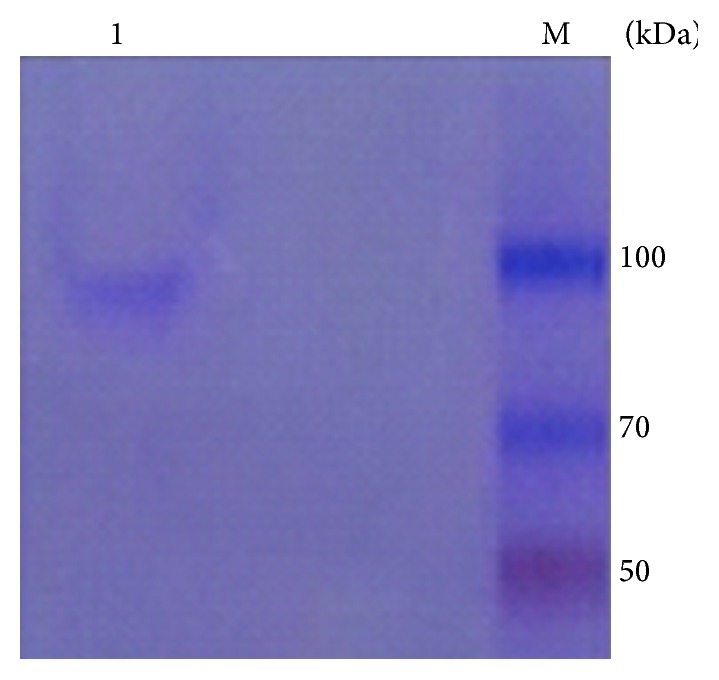
The SDS-PAGE. Line 1: hydroxylase; M: low molecular weight marker.

Table 1 summarizes the purification steps employed to purify the cholesterol hydroxylase. The TLC plate showed a deep blue spot that indicated that the enzyme had transformed cholesterol into 7*β*-hydroxyl cholesterol (Figure 3). The enzyme had a specific activity of 37.2 U/mg of protein at 30°C and pH 7.0. The retention time of 7*β*-hydroxycholesterol was 7.33 min. The standard curve is in Figure S16. The purified enzyme preparation gave a single band upon analysis by sodium dodecyl sulfate-polyacrylamide gel electrophoresis (SDS-PAGE) (Figure 5). Its molecular mass was estimated to be 80 kDa.

### 3.5. Amino Acid Sequence Analysis

The amino acid sequence of the purified enzyme was analyzed by MALDI-TOF/TOF. The MS data of pure enzyme is seen in Figure S17 and the MS/MS data of oligopeptides (Fr1–Fr3) in Figure S18 and those of oligopeptides (Fr4–Fr7) are seen in Figure S19. A trypsin digest of hydroxylase generated oligopeptides and the amino acid sequences of seven peptides (Fr1–Fr7) of the enzyme were determined. The sequences of Fr1–Fr7 were DWWPNQLNLNILHR, RFYENPAEFADAFAR, FYENPAEFADAFAR, HLFSYEW ELTK, IWLELSGGPNSR, VMNLDRFDLA, and SPAGAHQWVAK, respectively. These sequences are similar to those of catalases/peroxidases, for example, the catalase/peroxidase (*Burkholderia vietnamiensis* AU4i; the MS/MS data with Mascot accession number gi|543285001, protein sequence coverage: 24%), the heme catalase/peroxidase (*Burkholderia lata*, MS/MS data with Mascot accession number gi|78065290, 18%), catalase/hydroperoxidase HPI(I) (*Burkholderia cenocepacia*, MS/MS data with Mascot accession number gi|69880176, 12%), and catalase/hydroperoxidase HPI(I) (*Burkholderia ambifaria*, MS/MS data with Mascot accession number gi|493812374, 11%) (http://www.matrixscience.com/cgi/master_results.pl?file=../data/20130930/FTntOicSm.dat#Hit1).

## 4. Discussion

In the present study, the morphology of strain SE-1 showed different colony characteristics when grown on different media. It was pale yellow on the Beef Extract Peptone medium but was white and smaller in size on the screening medium. Doukyu reported that* Burkholderia cepacia* strain ST-200 produces an extracellular cholesterol oxidase which is stable and highly active in the presence of organic solvents. This cholesterol oxidase produces 6*β*-hydroperoxycholest-4-en-3-one from cholesterol. This oxidase contained bound flavin and was categorized as a 3*β*-hydroxysteroid oxidase, converting 3*β*-hydroxyl groups to keto groups. The molecular mass was 60 kDa. The enzyme is not inducible by cholesterol [20]. Under different nutritional conditions,* Burkholderia cepacia* strains can metabolize cholesterol by different pathways. Bacteria and fungi, especially of the genus of* Fusarium* and* Mucor*, that hydroxylate steroids at the C-7 position have been found. Kolek reported that dehydroepiandrosterone (DHEA), 5-androsten-3*β*,17*β*-diol, and 17*α*-methyl-5-androsten-3*β*,17*β*-diol were hydroxylated entirely at the 7*α*-axial, allylic position by* Fusarium culmorum*, which showed 7*α*-hydroxylase activity [21].* Botryodiplodia malorum* showed the 7*β*-hydroxylation of DHEA and 15*β*,16*β*-methylene-dehydroepiandrosterone [22]. Morfin demonstrated that* Fusarium moniliforme* could almost entirely hydroxylate DHEA at the 7*α*-axial, allylic position but could not hydroxylate cholesterol [23]. It was confirmed that the 7*α*-hydroxylase was a cytochrome p450 enzyme (Cyp7b), which occurred in humans and animals [24]. This enzyme required NADP^+^ as a coenzyme. Our experiments showed a requirement for NAD^+^ in the generation of 7*β*-hydroxycholesterol. Appleby reported that they found cytochrome P450 in bacteria in 1967 [25]. Such proteins have oxidase activity, could catalyze some various exogenous substances, and converted into useful products to the microorganisms themselves. Cytochrome P450, as the terminal oxidase, mainly uses molecular oxygen, after one atom of oxygen combined with substrate, the other atom of oxygen, and hydrogen atoms to generate water provided by the NAD(P)H. This implied that our isolate,* Burkholderia cepacia* SE-1, contained hydroxylase.

## 5. Conclusions

Hydroxylation at position 7 of steroids with controlled stereoselectivity allows for the production of important pharmaceutical intermediates. Therefore, it was necessary that the microbial strains were screened which can transform steroids effectively. In our study, cholesterol and 16*α*,17*α*-epoxypregnenolone were transformed into useful products and hydroxylase was isolated from* Burkholderia cepacia* SE-1 successfully. These studies suggest that the steroid hydroxylase possesses 7*β*-site-selectivity and has latency value in production of the steroid medicine.

## Supplementary Material

The ^1^H and ^13^C NMR spectral data for the product **1** was as follows: 1H NMR δ (600 MHz, CDCl3) 0.69(3H, s), 1.05(3H, s). 0.86(3H, d, *J*=2.4 Hz), 0.87(3H, d, *J*=3.0 Hz), 0.93(3H, d, *J*=6.6 Hz), 1.15–1.58(26H, m), 2.05(1H, m), 2.30(1H, m), 3.54(H, m), 3.84(1H, dt, *J*=8.4, 2.4 Hz), 5.28(1H, t, *J*=2.4 Hz); ^13^C NMR δ(125 MHz, CDCl3): 36.9 (C-1), 31.6 (C-2), 71.4 (C-3), 41.7 (C-4), 143.5 (C-5), 125.5 (C-6), 73.4 (C-7), 40.9 (C-8), 48.3 (C-9), 36.4 (C-10), 21.l (C-11), 28.6 (C-12), 42.9 (C-13), 55.5 (C-14), 26.4 (C-15), 39.6 (C-16), 54.9 (C-17), 11.8 (C-18), 19.2 (C-19), 35.7 (C-20), 18.7 (C-21), 36.2 (C-22), 23.9 (C-23), 39.5 (C-24), 28.0 (C-25), 22.6 (C-26), 22.8 (C-27).
The ^1^H and ^13^C NMR spectrum data of compound 2 was as follows: 1H NMR δ (600 MHz, CDCI3) 0.68 (s, 3H), 0.86(d, 3H, *J*=2.4), 0.87(d, 3H, *J*=3.0) 0.92 (d, 3H, *J*=6.6) 1.12 (s, 3H), 5.69 (1H, s C-6); ^13^C NMR δ(125 MHz, CDCl3): 36.4 (C-1), 31.3 (C-2), 70.6 (C-3), 41.8 (C-4), 165.0 (C-5), 126.2 (C-6), 202.2 (C-7), 45.5 (C-8), 50.0 (C-9), 38.3 (C-10), 21.3 (C-11), 28.6 (C-12), 43.1 (C-13), 54.9 (C-14), 26.3 (C-15), 38.8 (C-16), 54.9 (C-17), 12.0 (C-18), 18.9 (C-19), 35.8 (C-20), 18.7 (C-21), 36.2 (C-22), 23.9 (C-23), 39.5 (C-24), 28.0 (C-25), 22.6(C-26), 22.8 (C-27).

## Figures and Tables

**Table 1:** Purification of the cholesterol 7*β*-hydroxylase from *Burkholderia cepacia* SE-1.

Step	Volume (mL)	Total protein^a^ (mg)	Total activity^b^ (U)	Specific activity (U/mg)	Purification (fold)	Yield (%)
Ammonium sulfate	10	35.77	456.50	12.76	1.0	100.00
DEAE-cellulose	20	15.86	218.21	13.76	1.1	47.80
Sephadex G-200	10	5.76	107.69	18.70	1.5	23.59
PAGE^c^	0.5	0.58	21.59	37.22	2.9	4.73

^a^Protein concentration was determined by the method of Bradford [16] with bovine serum albumin as the standard.

^b^Cholesterol 7*β*-hydroxylase activity was assayed by measuring the quantity of 7*β*-cholesterol.

^c^PAGE: polyacrylamide gel electrophoresis.
