# Supplementary material for: Biotransformation of Cholesterol and 16α,17α-Epoxypregnenolone and Isolation of Hydroxylase in Burkholderia cepacia SE-1
Source: Biomed Res Int. 2016 Jun 2;2016:5727631. doi: 10.1155/2016/5727631 (PMC4909919; doi:10.1155/2016/5727631)
Supplement: Supplementary file 1 — The 1H and 13C NMR spectral data for the product 1 was as follows: 1H NMR δ (600 MHz, CDCl3) 0.69(3H, s), 1.05(3H, s). 0.86(3H, d, J=2.4 Hz), 0.87(3H, d, J=3.0 Hz), 0.93(3H, d, J=6.6 Hz), 1.15–1.58(26H, m), 2.05(1H, m), 2.30(1H, m), 3.54(H, m), 3.84(1H, dt, J=8.4, 2.4 Hz), 5.28(1H, t, J=2.4 Hz); 13C NMR δ(125 MHz, CDCl3): 36.9 (C-1), 31.6 (C-2), 71.4 (C-3), 41.7 (C-4), 143.5 (C-5), 125.5 (C-6), 73.4 (C-7), 40.9 (C-8), 48.3 (C-9), 36.4 (C-10), 21.l (C-11), 28.6 (C-12), 42.9 (C-13), 55.5 (C-14), 26.4 (C-15), 39.6 (C-16), 54.9 (C-17), 11.8 (C-18), 19.2 (C-19), 35.7 (C-20), 18.7 (C-21), 36.2 (C-22), 23.9 (C-23), 39.5 (C-24), 28.0 (C-25), 22.6 (C-26), 22.8 (C-27). The 1H and 13C NMR spectrum data of compound 2 was as follows: 1H NMR δ (600 MHz, CDCI3) 0.68 (s, 3H), 0.86(d, 3H, J=2.4), 0.87(d, 3H, J=3.0) 0.92 (d, 3H, J=6.6) 1.12 (s, 3H), 5.69 (1H, s C-6); 13C NMR δ(125 MHz, CDCl3): 36.4 (C-1), 31.3 (C-2), 70.6 (C-3), 41.8 (C-4), 165.0 (C-5), 126.2 (C-6), 202.2 (C-7), 45.5 (C-8), 50.0 (C-9), 38.3 (C-10), 21.3 (C-11), 28.6 (C-12), 43.1 (C-13), 54.9 (C-14), 26.3 (C-15), 38.8 (C-16), 54.9 (C-17), 12.0 (C-18), 18.9 (C-19), 35.8 (C-20), 18.7 (C-21), 36.2 (C-22), 23.9 (C-23), 39.5 (C-24), 28.0 (C-25), 22.6(C-26), 22.8 (C-27). [file 5727631.f1.pdf]

Figure S1

ste-1 13C

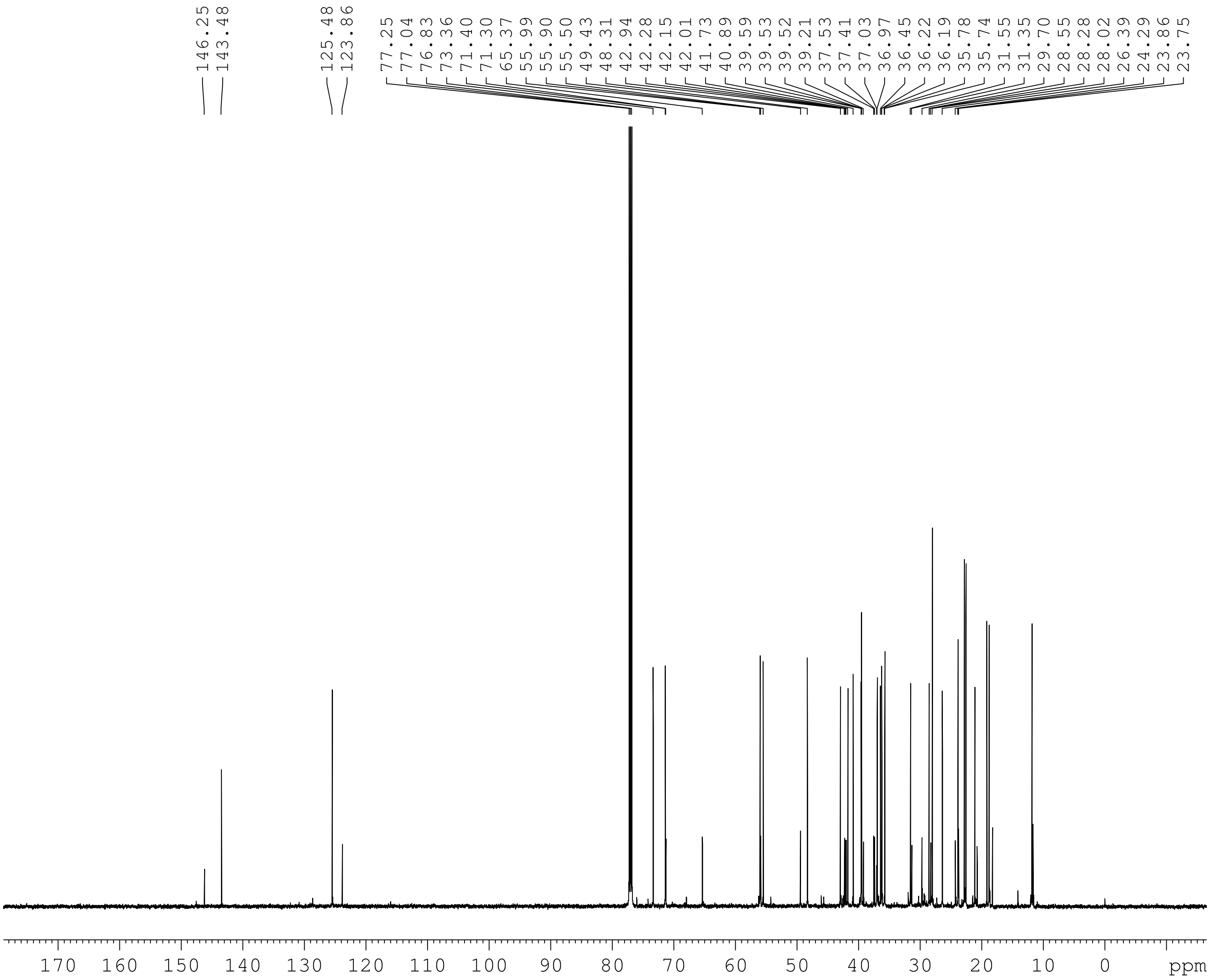

NAME	120380	
EXPNO	2	
PROCNO	1	
Date_	20120511	
Time	14.12	
INSTRUM	spect	
PROBHD	5 mm PABBO BB-	
PULPROG	zgpg30	
TD	65536	
SOLVENT	CDCl3	
NS	1616	
DS	4	
SWH	36057.691	Hz
FIDRES	0.550197	Hz
AQ	0.9088159	sec
RG	2050	
DW	13.867	usec
DE	6.50	usec
TE	298.4	K
D1	2.00000000	sec
D11	0.03000000	sec
TD0	1	

======== CHANNEL f1 ========

NUC1	13C	
P1	9.70	usec
PL1	1.00	dB
PL1W	79.21191406	W
SFO1	151.0310627	MHz

======== CHANNEL f2 ========

CPDPRG2	waltz16	
NUC2	1H	
PCPD2	80.00	usec
PL2	-2.00	dB
PL12	14.41	dB
PL13	13.65	dB
PL2W	23.13002586	W
PL12W	0.52865964	W
PL13W	0.62976158	W
SFO2	600.5824023	MHz
SI	65536	
SF	151.0159612	MHz
WDW	EM	
SSB	0	
LB	1.50	Hz
GB	0	
PC	1.40	

Figure S2

ste-1 DEPT

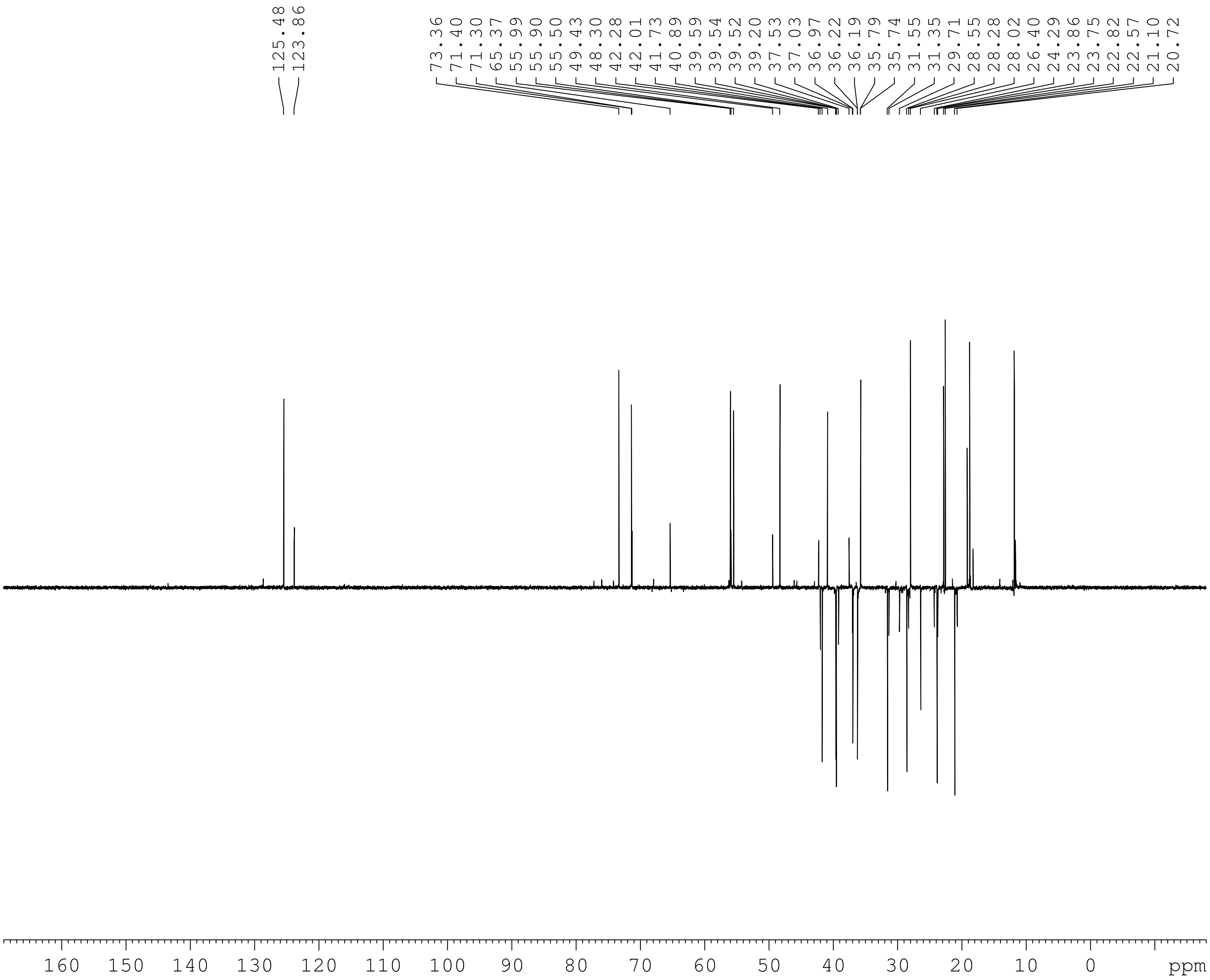

NAME	120380
EXPNO	3
PROCNO	1
Date_	20120511
Time	15.42
INSTRUM	spect
PROBHD	5 mm PABBO BB-
PULPROG	dept135
TD	65536
SOLVENT	CDCl3
NS	512
DS	4
SWH	36057.691 Hz
FIDRES	0.550197 Hz
AQ	0.9088159 sec
RG	2050
DW	13.867 usec
DE	6.50 usec
TE	298.0 K
CNST2	145.0000000
D1	2.00000000 sec
D2	0.00344828 sec
D12	0.00002000 sec
TD0	1

======== CHANNEL f1 ========

NUC1	13C
P1	9.70 usec
P2	19.40 usec
PL1	1.00 dB
PL1W	79.21191406 W
SFO1	151.0310627 MHz

======== CHANNEL f2 ========

CPDPRG2	waltz16
NUC2	1H
P3	12.10 usec
P4	24.20 usec
PCPD2	80.00 usec
PL2	-2.00 dB
PL12	14.41 dB
PL2W	23.13002586 W
PL12W	0.52865964 W
SFO2	600.5824023 MHz
SI	65536
SF	151.0159610 MHz
WDW	EM
SSB	0
LB	1.00 Hz
GB	0
PC	1.40

Figure S3

ste-1

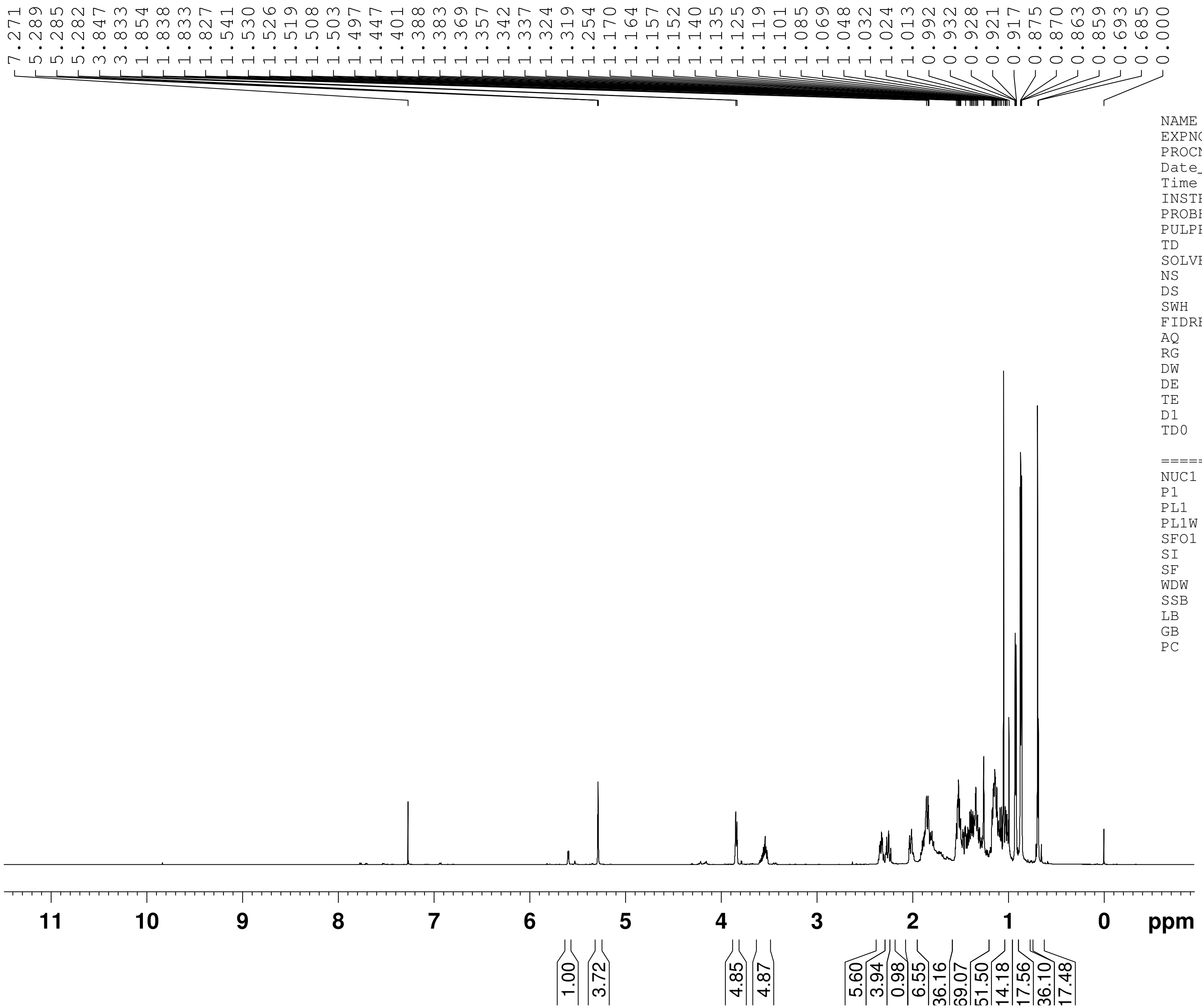

NAME	120380
EXPNO	1
PROCNO	1
Date_	20120511
Time	14.09
INSTRUM	spect
PROBHD	5 mm PABBO BB-
PULPROG	zg30
TD	65536
SOLVENT	CDCl3
NS	32
DS	2
SWH	9615.385 Hz
FIDRES	0.146719 Hz
AQ	3.4079220 sec
RG	32
DW	52.000 usec
DE	6.50 usec
TE	297.9 K
D1	1.00000000 sec
TD0	1

======== CHANNEL f1 ========

NUC1	1H
P1	12.10 usec
PL1	-2.00 dB
PL1W	23.13002586 W
SFO1	600.5840839 MHz
SI	65536
SF	600.5800118 MHz
WDW	EM
SSB	0
LB	0.40 Hz
GB	0
PC	1.00

Figure S4

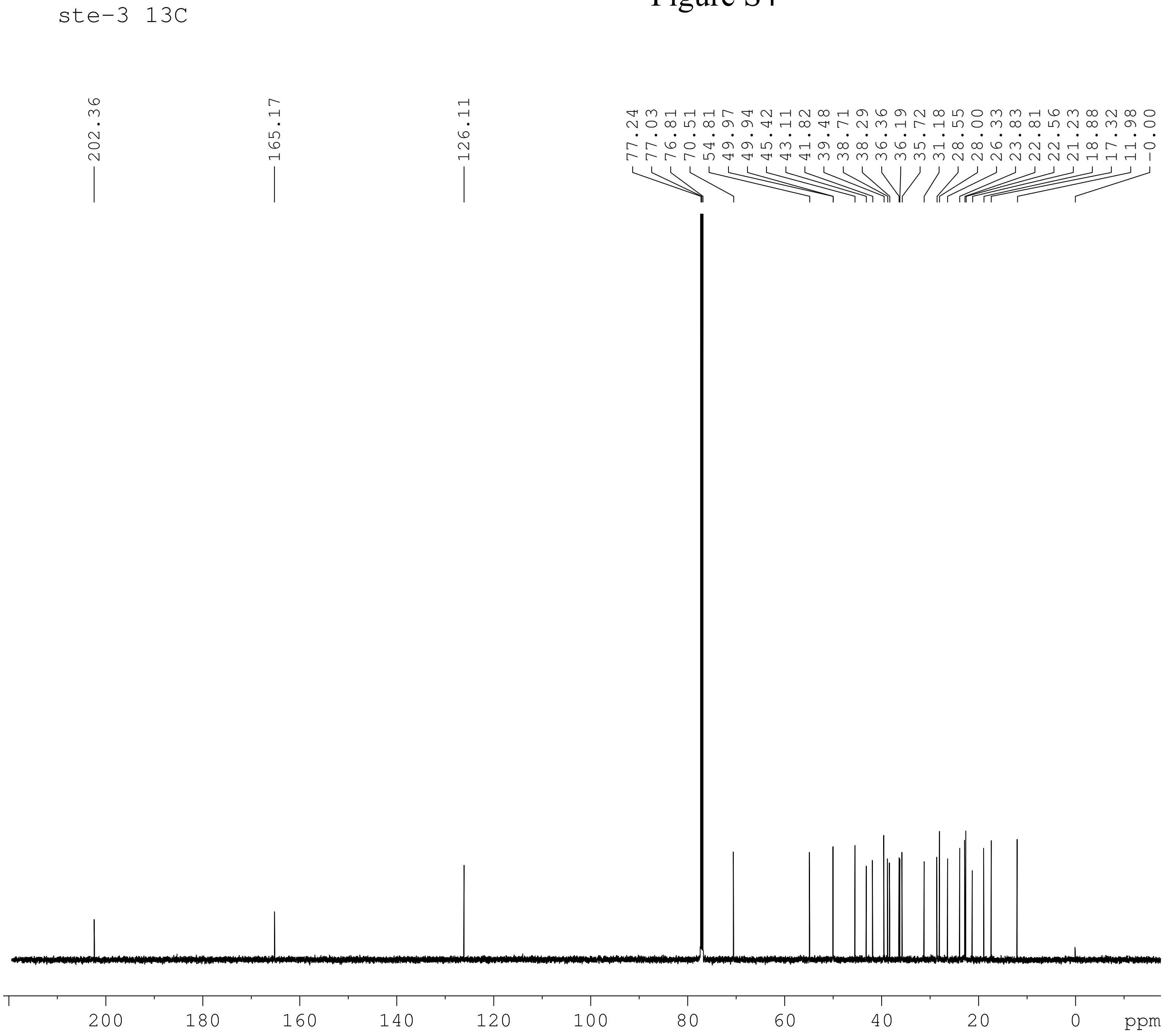

NAME	120709	
EXPNO	2	
PROCNO	1	
Date_	20121010	
Time	10.40	
INSTRUM	spect	
PROBHD	5 mm PABBO BB-	
PULPROG	zgpg30	
TD	65536	
SOLVENT	CDCl3	
NS	408	
DS	4	
SWH	36057.691	Hz
FIDRES	0.550197	Hz
AQ	0.9088159	sec
RG	2050	
DW	13.867	usec
DE	6.50	usec
TE	295.0	K
D1	2.00000000	sec
D11	0.03000000	sec
TD0	1	
======== CHANNEL f1 ========		
NUC1	13C	
P1	9.70	usec
PL1	1.00	dB
PL1W	79.21191406	W
SFO1	151.0310627	MHz
======== CHANNEL f2 ========		
CPDPRG2	waltz16	
NUC2	1H	
PCPD2	80.00	usec
PL2	-2.00	dB
PL12	14.41	dB
PL13	13.65	dB
PL2W	23.13002586	W
PL12W	0.52865964	W
PL13W	0.62976158	W
SFO2	600.5824023	MHz
SI	65536	
SF	151.0159624	MHz
WDW	EM	
SSB	0	
LB	1.00	Hz
GB	0	
PC	1.40	

Figure S5

ste-2 DEPT135

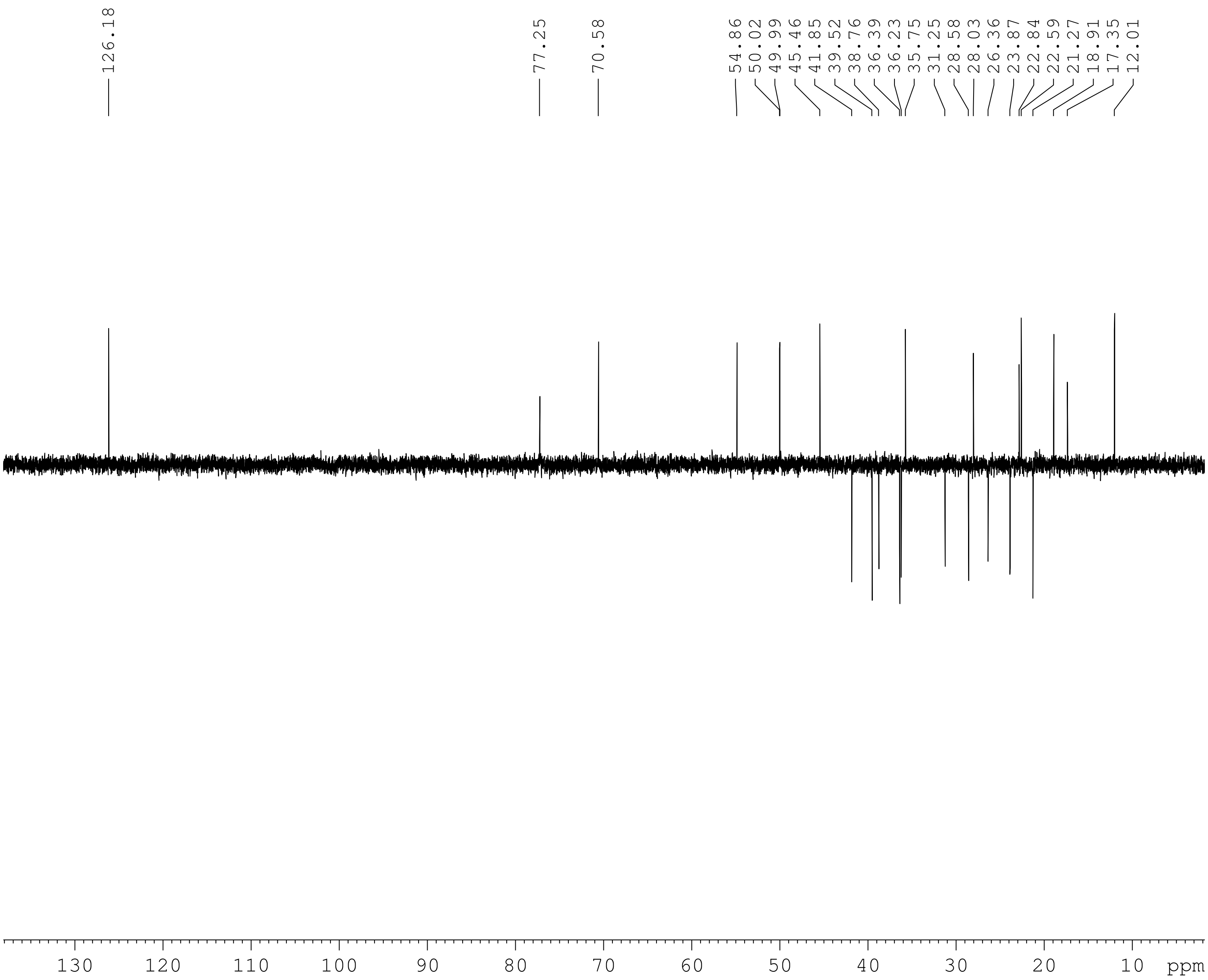

NAME	120503
EXPNO	3
PROCNO	1
Date_	20120618
Time	14.07
INSTRUM	spect
PROBHD	5 mm PABBO BB-
PULPROG	dept135
TD	65536
SOLVENT	CDCl3
NS	1600
DS	8
SWH	36057.691 Hz
FIDRES	0.550197 Hz
AQ	0.9088159 sec
RG	2050
DW	13.867 usec
DE	6.50 usec
TE	295.9 K
CNST2	145.0000000
D1	2.00000000 sec
D2	0.00344828 sec
D12	0.00002000 sec
TD0	1

======== CHANNEL f1 ========

NUC1	13C
P1	9.70 usec
P2	19.40 usec
PL1	1.00 dB
PL1W	79.21191406 W
SFO1	151.0310626 MHz

======== CHANNEL f2 ========

CPDPRG2	waltz16
NUC2	1H
P3	12.10 usec
P4	24.20 usec
PCPD2	80.00 usec
PL2	-2.00 dB
PL12	14.41 dB
PL2W	23.13002586 W
PL12W	0.52865964 W
SFO2	600.5824023 MHz
SI	65536
SF	151.0159570 MHz
WDW	EM
SSB	0
LB	1.00 Hz
GB	0
PC	1.40

Figure S6

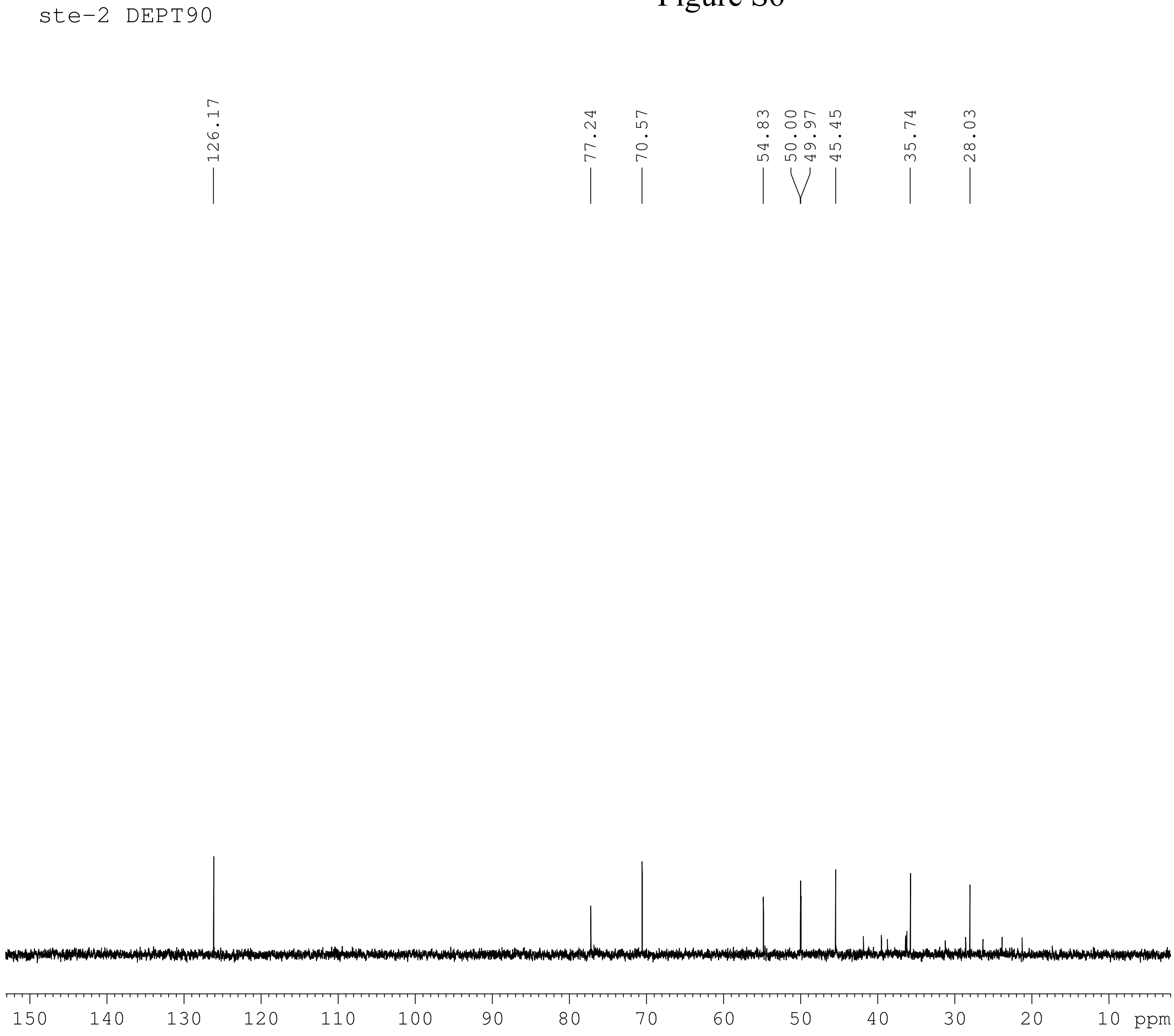

NAME	120503
EXPNO	4
PROCNO	1
Date_	20120618
Time	12.48
INSTRUM	spect
PROBHD	5 mm PABBO BB-
PULPROG	dept90
TD	65536
SOLVENT	CDCl3
NS	1600
DS	8
SWH	36057.691 Hz
FIDRES	0.550197 Hz
AQ	0.9088159 sec
RG	2050
DW	13.867 usec
DE	6.50 usec
TE	295.8 K
CNST2	145.0000000
D1	2.00000000 sec
D2	0.00344828 sec
D12	0.00002000 sec
TD0	1
======== CHANNEL f1 ========	
NUC1	13C
P1	9.70 usec
P2	19.40 usec
PL1	1.00 dB
PL1W	79.21191406 W
SFO1	151.0310627 MHz
======== CHANNEL f2 ========	
CPDPRG2	waltz16
NUC2	1H
P3	12.10 usec
P4	24.20 usec
PCPD2	80.00 usec
PL2	-2.00 dB
PL12	14.41 dB
PL2W	23.13002586 W
PL12W	0.52865964 W
SFO2	600.5824023 MHz
SI	32768
SF	151.0159584 MHz
WDW	EM
SSB	0
LB	2.00 Hz
GB	0
PC	1.40

Figure S7

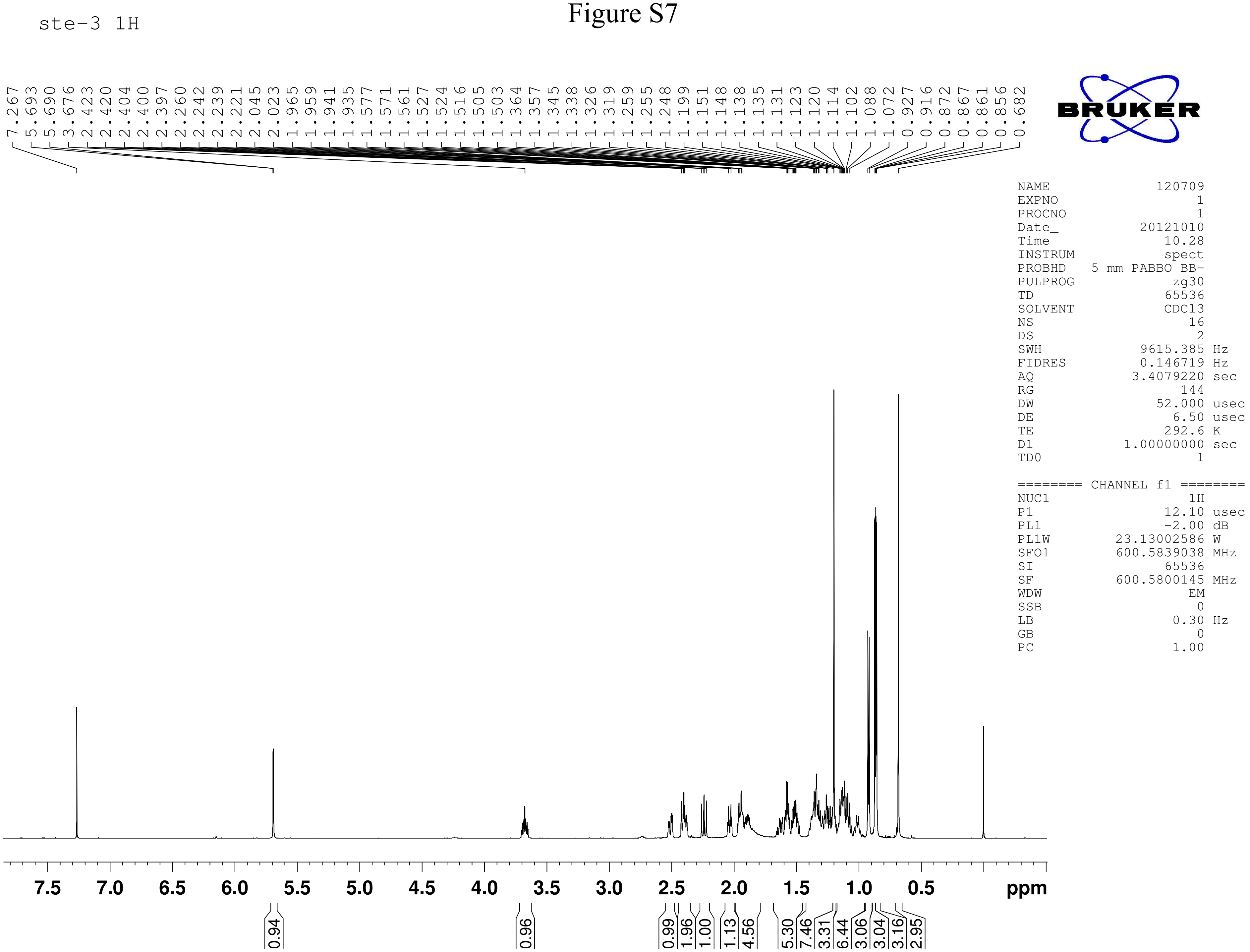
ste-3 1H
7.267
5.693
5.690
3.676
2.423
2.420
2.404
2.400
2.397
2.260
2.242
2.239
2.221
2.045
2.023
1.965
1.959
1.941
1.935
1.577
1.571
1.561
1.527
1.524
1.516
1.505
1.503
1.364
1.357
1.345
1.338
1.326
1.319
1.259
1.255
1.248
1.199
1.151
1.148
1.138
1.135
1.131
1.123
1.120
1.114
1.102
1.088
1.072
0.927
0.916
0.872
0.867
0.861
0.856
0.682
BRUKER
NAME 120709
EXPNO 1
PROCNO 1
Date_ 20121010
Time 10.28
INSTRUM spect
PROBHD 5 mm PABBO BB-
PULPROG zg30
TD 65536
SOLVENT CDCl3
NS 16
DS 2
SWH 9615.385 Hz
FIDRES 0.146719 Hz
AQ 3.4079220 sec
RG 144
DW 52.000 usec
DE 6.50 usec
TE 292.6 K
D1 1.00000000 sec
TD0 1
======== CHANNEL f1 ========
NUC1 1H
P1 12.10 usec
PL1 -2.00 dB
PL1W 23.13002586 W
SFO1 600.5839038 MHz
SI 65536
SF 600.5800145 MHz
WDW EM
SSB 0
LB 0.30 Hz
GB 0
PC 1.00
7.5
7.0
6.5
6.0
5.5
5.0
4.5
4.0
3.5
3.0
2.5
2.0
1.5
1.0
0.5
ppm
0.94
0.96
0.99
1.96
1.00
1.13
4.56
5.30
7.46
3.31
6.44
3.06
3.04
3.16
2.95

Figure S8

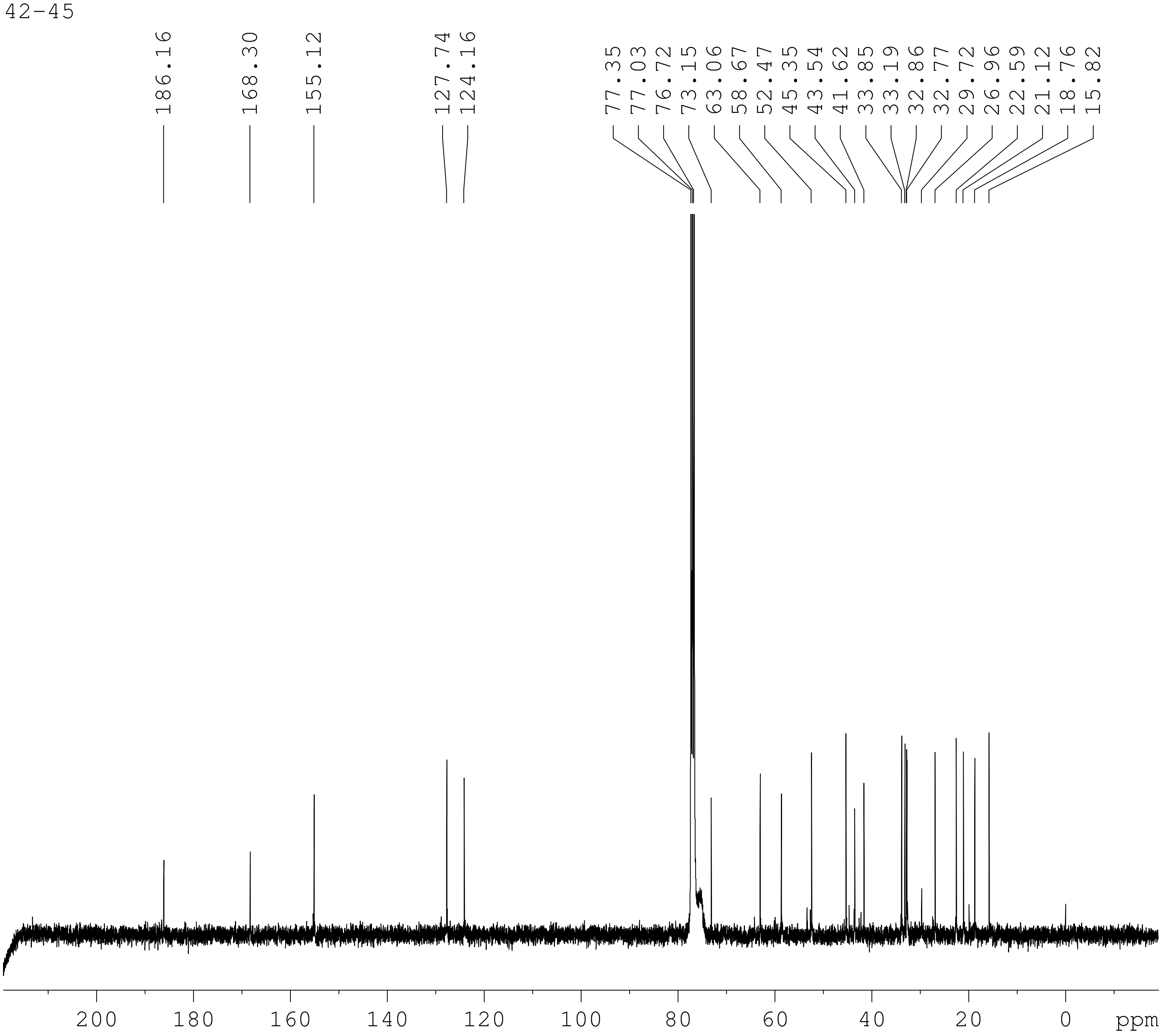


```
Current Data Parameters
NAME             zhongkai
EXPNO                  14
PROCNO                  1

F2 - Acquisition Parameters
Date_            20140815
Time                18.35
INSTRUM             spect
PROBHD   5 mm BBO BB-1H
PULPROG           zgpg30
TD                 65536
SOLVENT            CDCl3
NS                 15194
DS                     0
SWH           23980.814 Hz
FIDRES         0.365918 Hz
AQ            1.3664756 sec
RG                  8192
DW                20.850 usec
DE                  6.00 usec
TE                 300.1 K
D1            2.00000000 sec
d11           0.03000000 sec
MCREST        0.00000000 sec
MCWRK         0.01500000 sec

======== CHANNEL f1 ========
NUC1                 13C
P1                  8.00 usec
PL1                -2.00 dB
SFO1        100.6228298 MHz

======== CHANNEL f2 ========
CPDPRG2          waltz16
NUC2                  1H
PCPD2              80.00 usec
PL2                -3.00 dB
PL12                1.08 dB
PL13                1.08 dB
SFO2        400.1316005 MHz

F2 - Processing parameters
SI                 32768
SF          100.6127613 MHz
WDW                   EM
SSB                    0
LB                  1.00 Hz
GB                     0
PC                  1.40
```

Figure S9

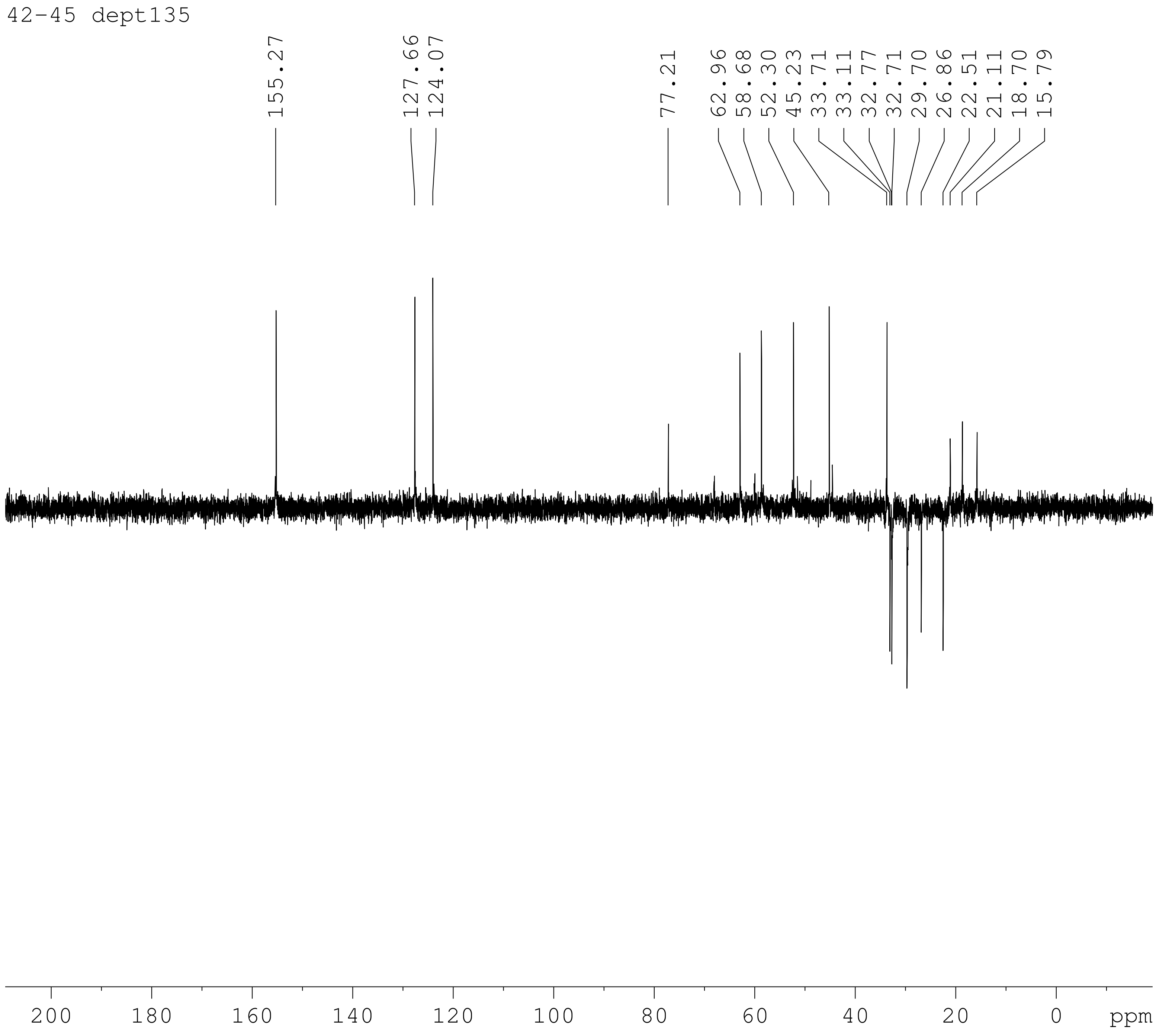

Current Data Parameters
NAME zhangzhibin
EXPNO 224
PROCNO 1

F2 - Acquisition Parameters
Date_ 20141110
Time 8.35
INSTRUM spect
PROBHD 5 mm BBO BB-1H
PULPROG dept135
TD 65536
SOLVENT CDCl3
NS 11004
DS 4
SWH 23980.814 Hz
FIDRES 0.365918 Hz
AQ 1.3664756 sec
RG 16384
DW 20.850 usec
DE 6.00 usec
TE 297.5 K
CNST2 145.0000000
D1 2.00000000 sec
d2 0.00344828 sec
d12 0.00002000 sec
DELTA 0.00001019 sec
MCREST 0.00000000 sec
MCWRK 0.01500000 sec

======== CHANNEL f1 ========
NUC1 13C
P1 8.00 usec
p2 16.00 usec
PL1 -2.00 dB
SFO1 100.6228298 MHz

======== CHANNEL f2 ========
CPDPRG2 waltz16
NUC2 1H
P3 14.00 usec
p4 28.00 usec
PCPD2 80.00 usec
PL2 -3.00 dB
PL12 12.14 dB
SFO2 400.1316005 MHz

F2 - Processing parameters
SI 32768
SF 100.6127690 MHz
WDW EM
SSB 0
LB 1.00 Hz
GB 0
PC 1.40

Figure S10

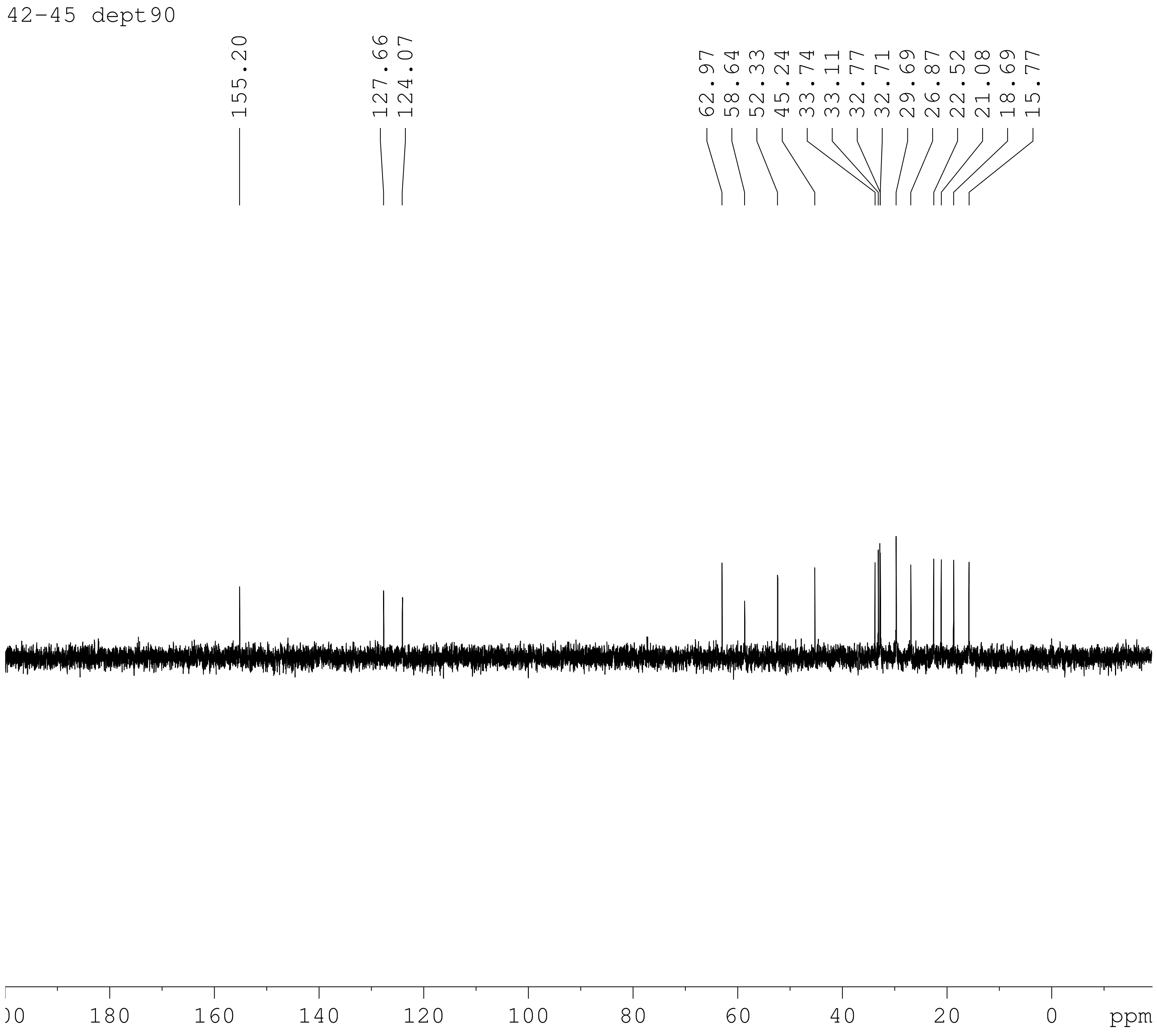


```
Current Data Parameters
NAME              zhongkai
EXPNO                   19
PROCNO                   1

F2 - Acquisition Parameters
Date_             20140828
Time                 22.57
INSTRUM              spect
PROBHD   5 mm BBO BB-1H
PULPROG             dept90
TD                   65536
SOLVENT              CDCl3
NS                   16532
DS                       4
SWH              23980.814 Hz
FIDRES            0.365918 Hz
AQ               1.3664756 sec
RG                   16384
DW                  20.850 usec
DE                    6.00 usec
TE                   299.3 K
CNST2          145.0000000
D1              2.00000000 sec
d2              0.00344828 sec
d12             0.00002000 sec
DELTA           0.00001019 sec
MCREST          0.00000000 sec
MCWRK           0.01500000 sec

======== CHANNEL f1 ========
NUC1                   13C
P1                    8.00 usec
p2                   16.00 usec
PL1                  -2.00 dB
SFO1           100.6228298 MHz

======== CHANNEL f2 ========
CPDPRG2            waltz16
NUC2                    1H
P3                   14.00 usec
p4                   28.00 usec
PCPD2                80.00 usec
PL2                  -3.00 dB
PL12                  1.08 dB
SFO2           400.1316005 MHz

F2 - Processing parameters
SI                   32768
SF             100.6127690 MHz
WDW                     EM
SSB                      0
LB                    1.00 Hz
GB                       0
PC                    1.40
```

Figure S11

42-45 HMQC

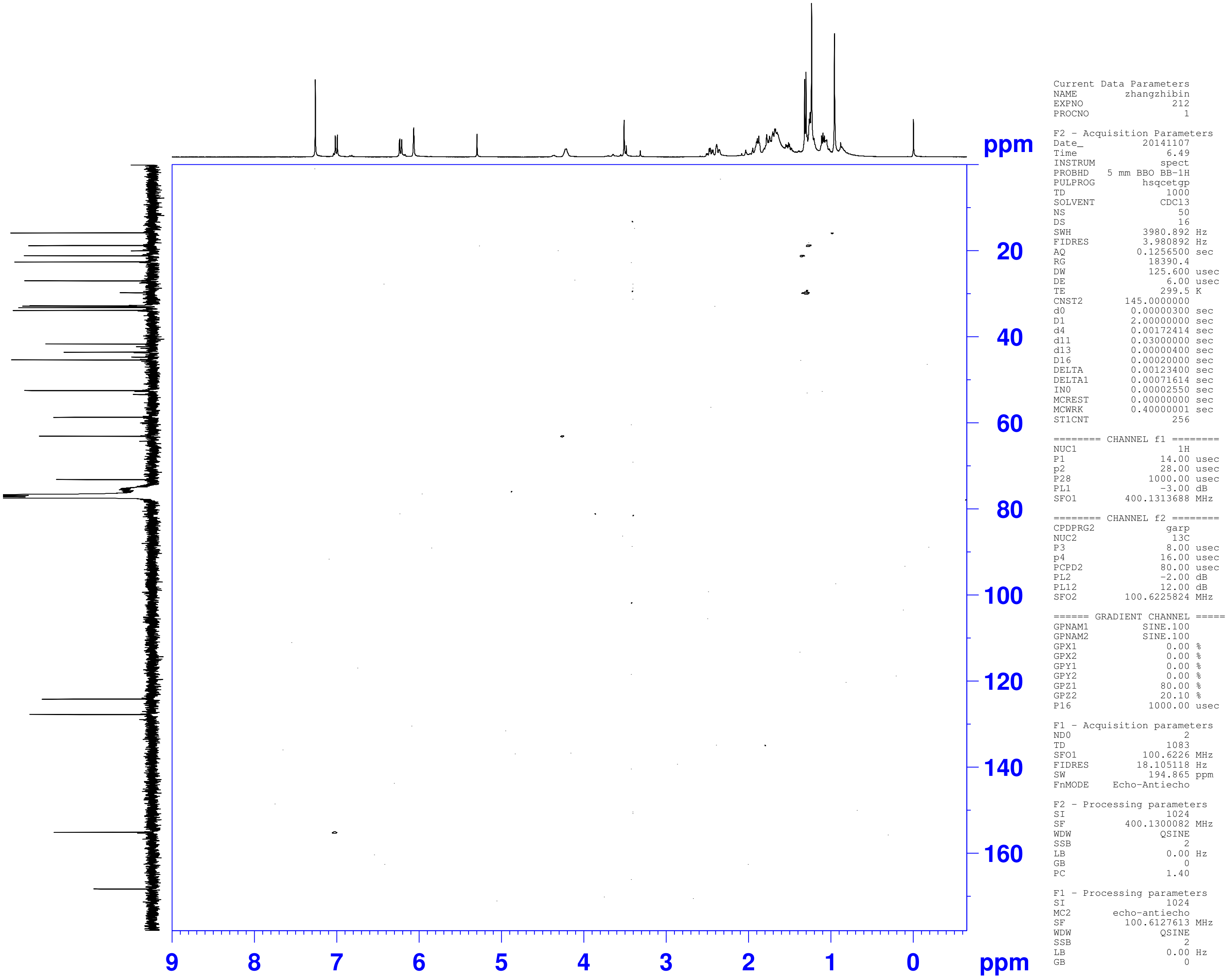

Figure S12

42-45 HMBC

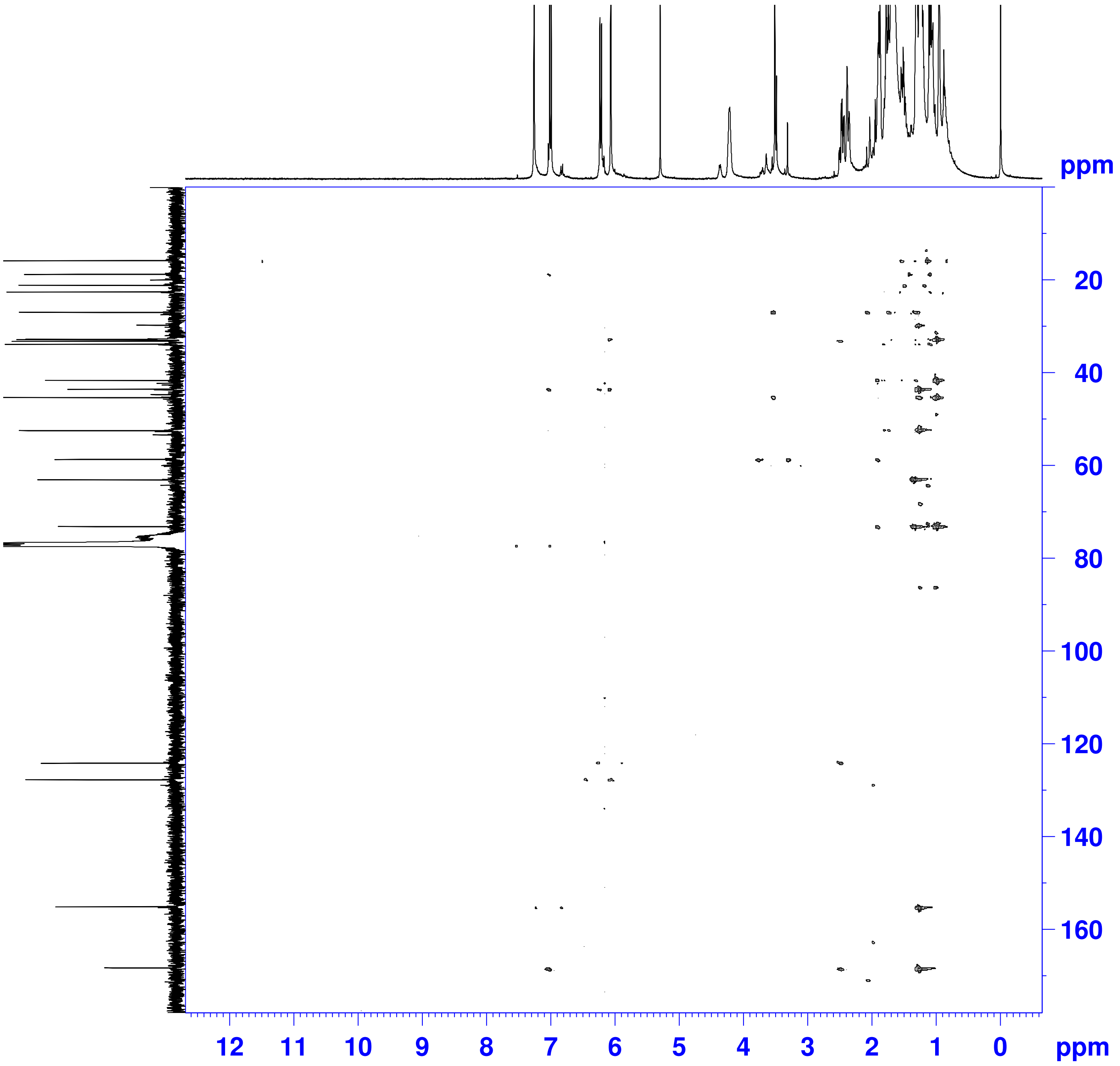


```
Current Data Parameters
NAME            zhongkai
EXPNO                 26
PROCNO                 1

F2 - Acquisition Parameters
Date_           20141121
Time               16.28
INSTRUM            spect
PROBHD   5 mm BBO BB-1H
PULPROG       hmbcgpndqf
TD                  4096
SOLVENT            CDCl3
NS                   128
DS                    16
SWH             4280.822 Hz
FIDRES          1.045123 Hz
AQ             0.4784628 sec
RG                 26008
DW               116.800 usec
DE                  6.00 usec
TE                 295.6 K
CNST13         8.0000000
d0            0.00000300 sec
D1            1.50000000 sec
d6            0.06250000 sec
D16           0.00020000 sec
IN0           0.00002481 sec
MCREST        0.00000000 sec
MCWRK         1.50000000 sec

======== CHANNEL f1 ========
NUC1                  1H
P1                 14.00 usec
p2                 28.00 usec
PL1                -3.00 dB
SFO1         400.1324688 MHz

======== CHANNEL f2 ========
NUC2                 13C
P3                  8.00 usec
PL2                -2.00 dB
SFO2         100.6228298 MHz

====== GRADIENT CHANNEL =====
GPNAM1          SINE.100
GPNAM2          SINE.100
GPNAM3          SINE.100
GPX1                0.00 %
GPX2                0.00 %
GPX3                0.00 %
GPY1                0.00 %
GPY2                0.00 %
GPY3                0.00 %
GPZ1               50.00 %
GPZ2               30.00 %
GPZ3               40.10 %
P16              1000.00 usec

F1 - Acquisition parameters
ND0                    2
TD                   732
SFO1            100.6228 MHz
FIDRES         27.528872 Hz
SW               200.264 ppm
FnMODE                QF

F2 - Processing parameters
SI                  1024
SF           400.1300082 MHz
WDW                 SINE
SSB                    0
LB                  0.00 Hz
GB                     0
PC                  1.40

F1 - Processing parameters
SI                  1024
MC2                   QF
SF           100.6127613 MHz
WDW                 SINE
SSB                    0
LB                  0.00 Hz
GB                     0
```

Figure S13

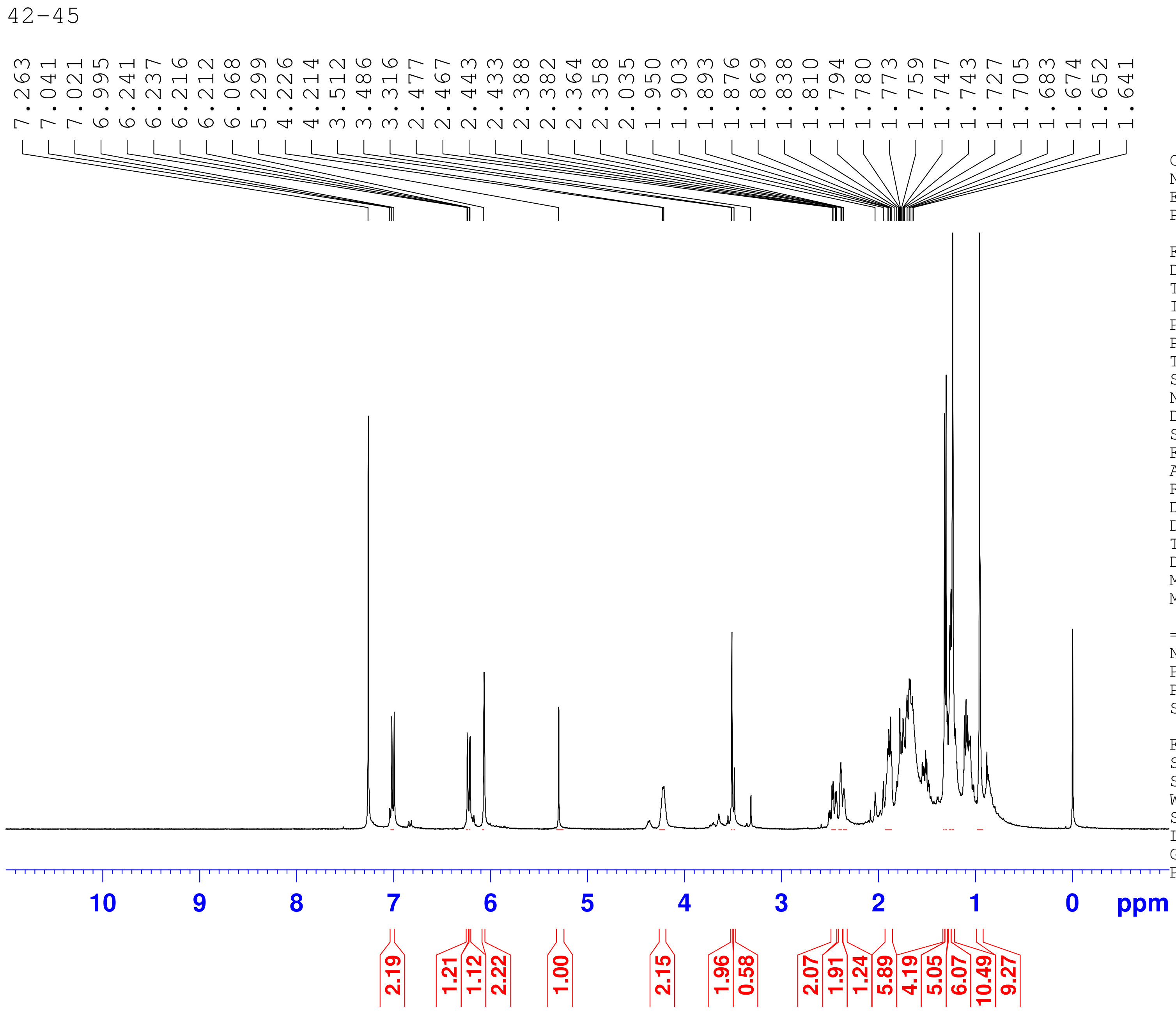

Current Data Parameters
NAME zhongkai
EXPNO 13
PROCNO 1

F2 - Acquisition Parameters
Date_ 20140815
Time 13.27
INSTRUM spect
PROBHD 5 mm BBO BB-1H
PULPROG zg30
TD 65536
SOLVENT CDCl3
NS 1528
DS 0
SWH 8278.146 Hz
FIDRES 0.126314 Hz
AQ 3.9584243 sec
RG 1625.5
DW 60.400 usec
DE 6.00 usec
TE 298.1 K
D1 1.00000000 sec
MCREST 0.00000000 sec
MCWRK 0.01500000 sec

======== CHANNEL f1 ========
NUC1 1H
P1 14.00 usec
PL1 -3.00 dB
SFO1 400.1324710 MHz

F2 - Processing parameters
SI 32768
SF 400.1300082 MHz
WDW EM
SSB 0
LB 0.30 Hz
GB 0
PC 1.00

Figure S14: The ^{1}H spectral data for compound 1-3

The ^{1}H and ^{13}C NMR spectral data for compound 1 was as follows: ^{1}H NMR • (600 MHz, $CDCl_3$) 0.69(3H, s), 1.05(3H,s)，0.86(3H, d, J=2.4 Hz), 0.87(3H,d,J＝3.0 Hz), 0.93(3H, d, J=6.6 Hz), 1.15-1.58(26H, m), 2.05(1H, m), 2.30(1H, m), 3.54(H, m), 3.84(1H, dt, J=8.4, 2.4 Hz)，5.28(1H, t, J＝2.4 Hz).

The 1H and 13C NMR spectrum data of compound **2** was as follows: ^{1}H NMR • (600MHz, CDCl3) 0.68（s，3H），0.86(d，3H，J=2.4)，0.87(d，3H，J=3.0)，0.92（d，3H，J=6.6），1.12（s，3H），5.69（1H，s，C-6.

The ^{1}H and ^{13}C NMR spectrum data of compound 3 was as follows: ^{1}H NMR • (400MHz, $CDCl_3$) , 7.01(1H, d ,J=10.4Hz), 6.22(1H, dd, J=10.4,1.6Hz), 6.06(1H, s), 4.22(1H, q, J=4.8Hz) , 3.51(1H, d, s),1.30(3H, d, J=6.4Hz), 1.23(3H, s), 0.95(3H, s); ^{13}C NMR •(100MHz, $CDCl_3$): 155.1 (C-1), 127.7 (C-2), 186.1 (C-3), 124.1 (C-4), 168.3 (C-5), 33.1 (C-6), 32.7 (C-7), 33.7(C-8),52.3 (C-9), 45.2 (C-10), 22.5 (C-11), 32.7 (C-12), 41.6 (C-13), 43.5 (C-14), 29.7 (C-15), 58.6 (C-16),73.1 (C-17), 15.7 (C-18), 21.1(C-19),62.9 (C-20), 18.7 (C-21).

Figure S15: The ^{13}C NMR spectral data for compound 1-3

C	(*1*)	(*2*)	(*3*)	*cholesterol*
1	36.9	36.4	155.1	37.3
2	31.6	31.3	127.7	31.6
3	71.4	70.6	186.1	71.6
4	41.7	41.8	124.1	42.2
5	143.5	165.0	168.3	140.8
6	125.5	126.2	33.1	121.6
7	73.4	202.2	32.7	31.9
8	40.9	45.5	33.7	31.9
9	48.3	50.0	52.3	50.2
10	36.4	38.3	45.2	36.5
11	21.1	21.3	22.5	21.1
12	28.6	28.6	32.7	28.3
13	42.9	43.1	41.6	42.3
14	55.5	54.9	43.5	56.8
15	26.4	26.3	29.7	24.3
16	39.6	38.8	58.6	39.8
17	54.9	54.9	73.1	56.8
18	11.8	12.0	15.7	11.9
19	19.2	18.9	21.1	19.4
20	35.7	35.8	62.9	35.8
21	18.7	18.7	18.7	18.7
22	36.2	36.2		36.2
23	23.9	23.9		23.9
24	39.5	39.5		39.5
25	28.0	28.0		28.0
26	22.6	22.6		22.6
27	22.8	22.8		22.8

Figure S16

Standard concentration 7 -hydroxycholesterol Preparation method

weight (ug)	Peak area (10^6)
3	1.368915
6	2.070902
9	2.579276
12	3.232314
15	4.027717

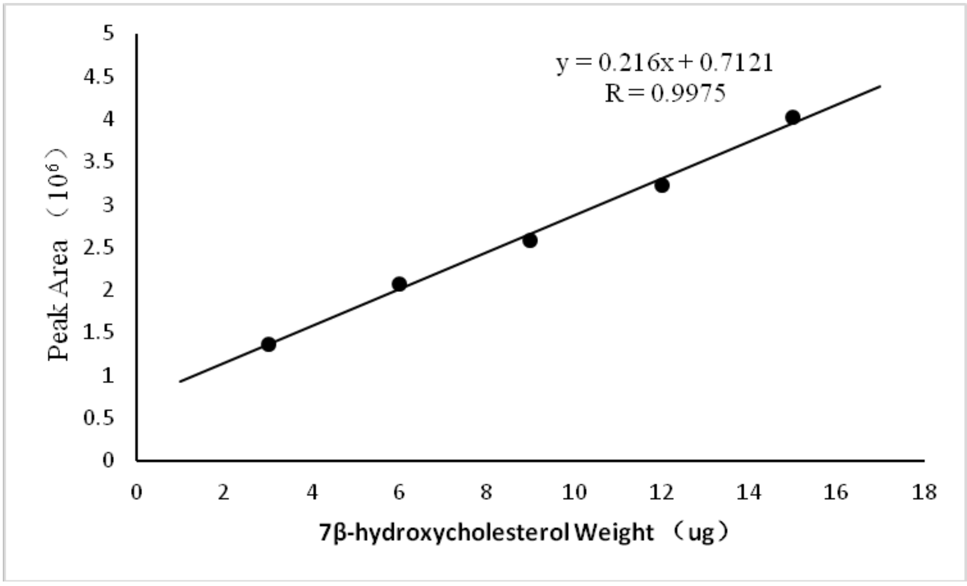

Standard curve of 7 -hydroxycholesterol

4700 Reflector Spec #1 MC[BP = 1647.8, 3647]

Figure S17

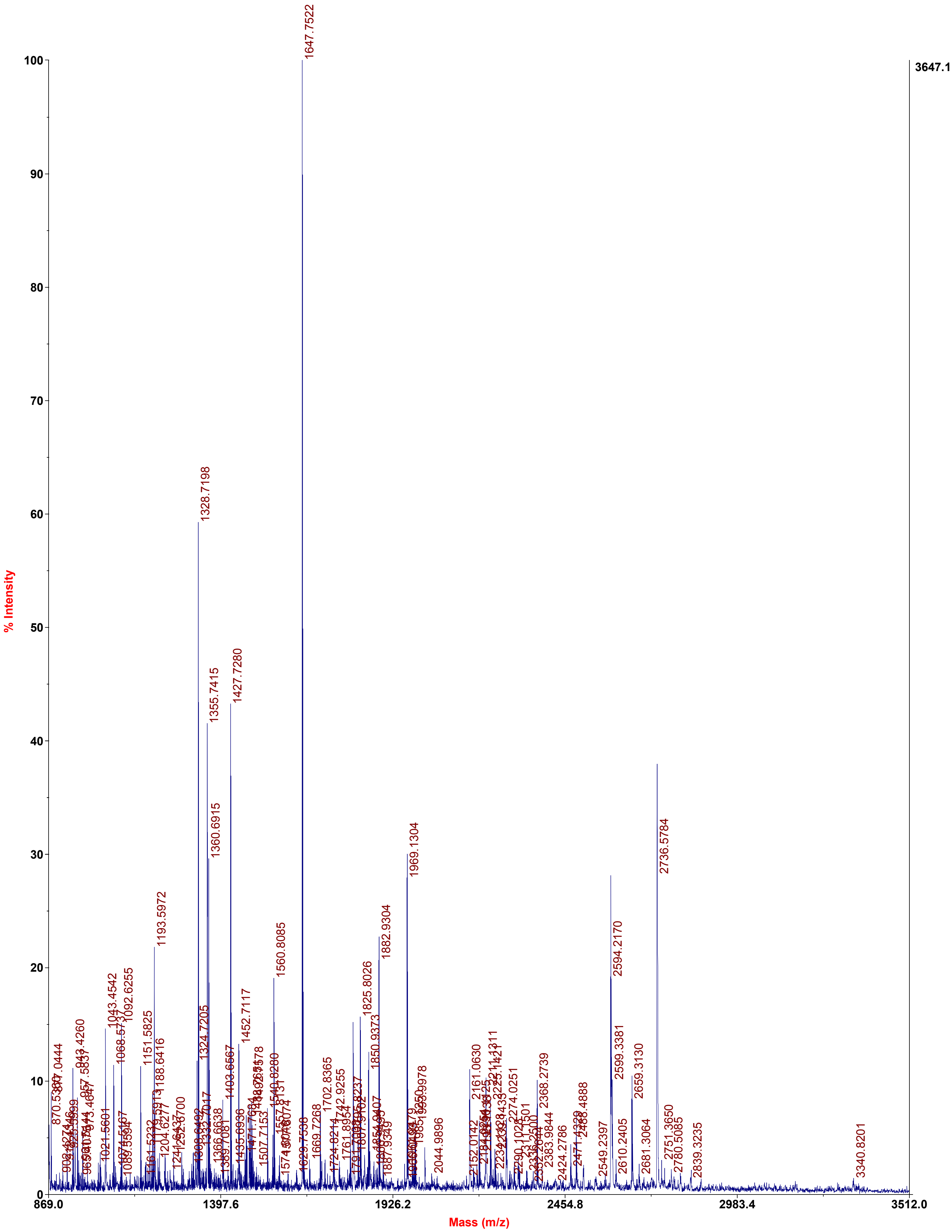

F:\...\J10_MS_11.t2d
Acquired:

Figure S18

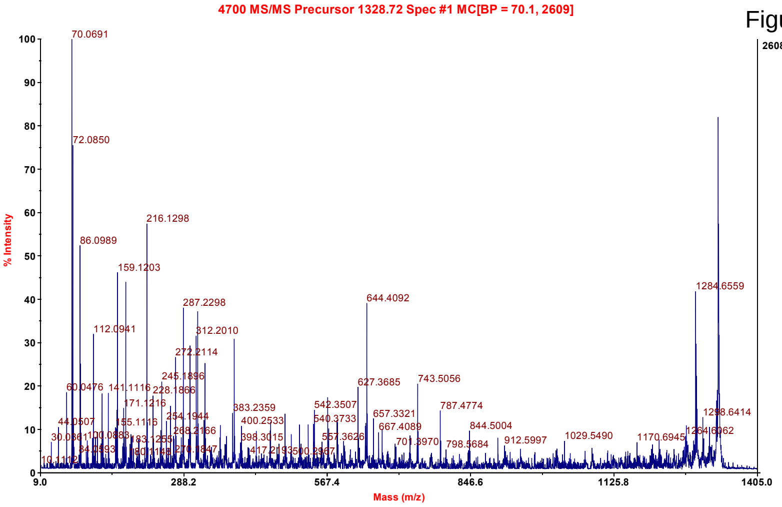

F:\...\J10_MSMS_1328.7202_109.t2d
Acquired:

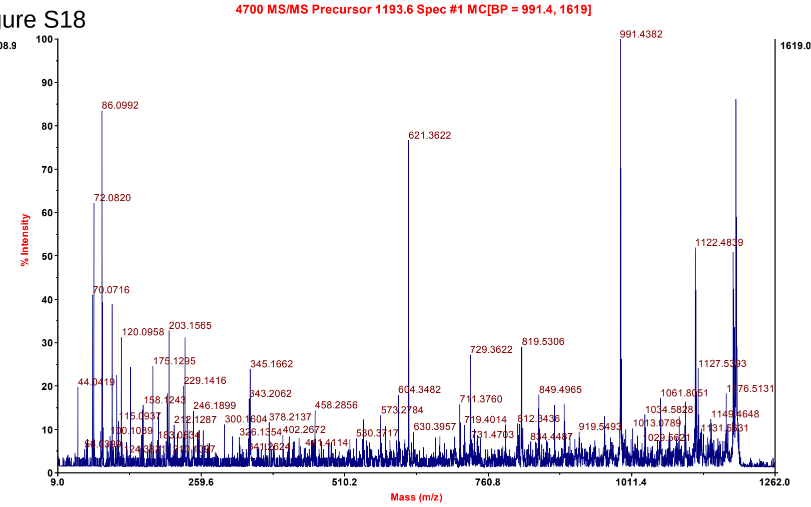

F:\...\J10_MSMS_1193.5967_100.t2d
Acquired:

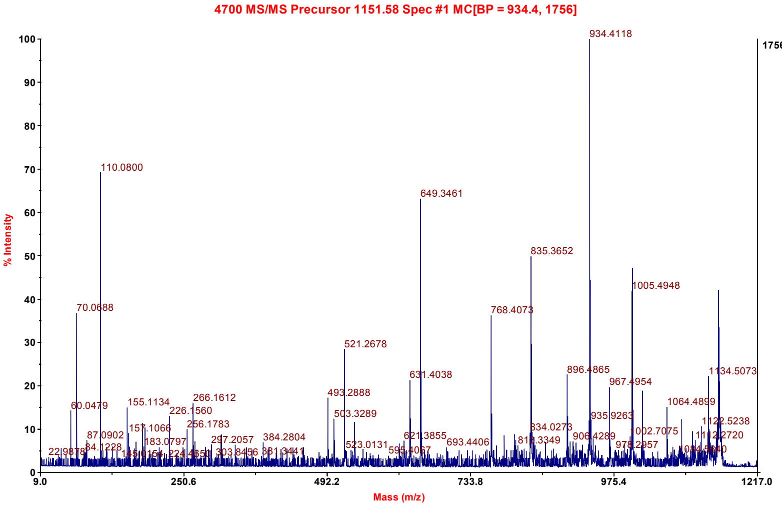

F:\...\J10_MSMS_1151.5807_90.t2d
Acquired:

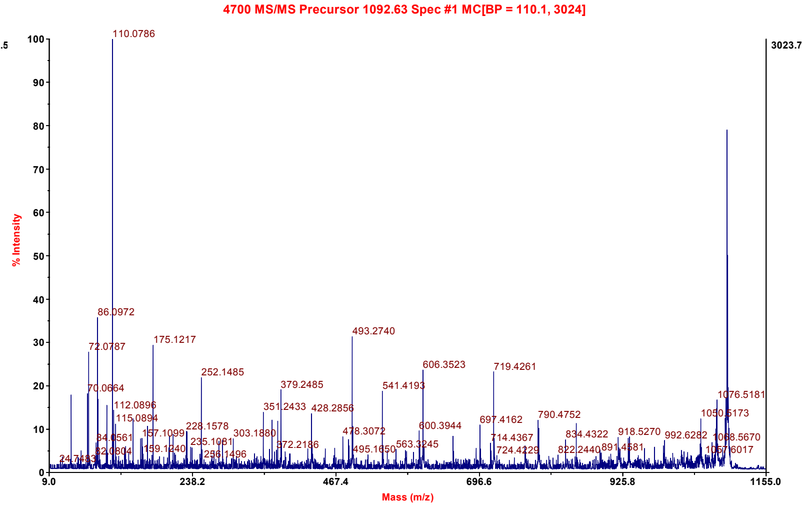

F:\...\J10_MSMS_1092.6276_91.t2d
Acquired:

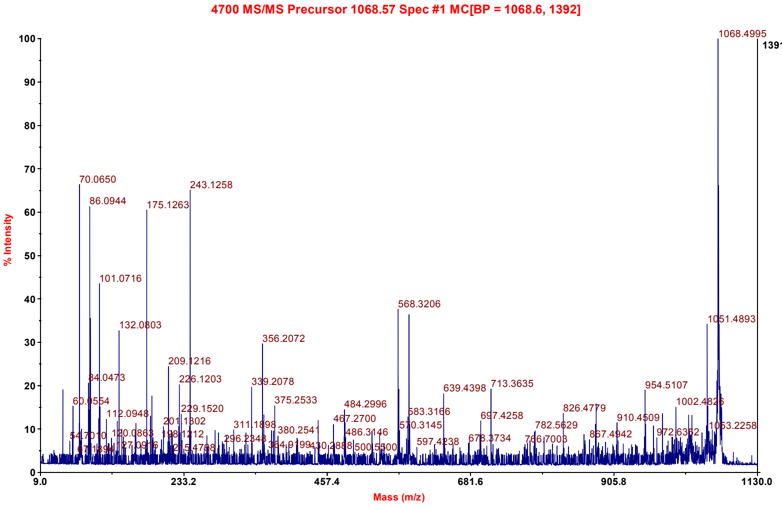

F:\...\J10_MSMS_1068.5743_87.t2d
Acquired:

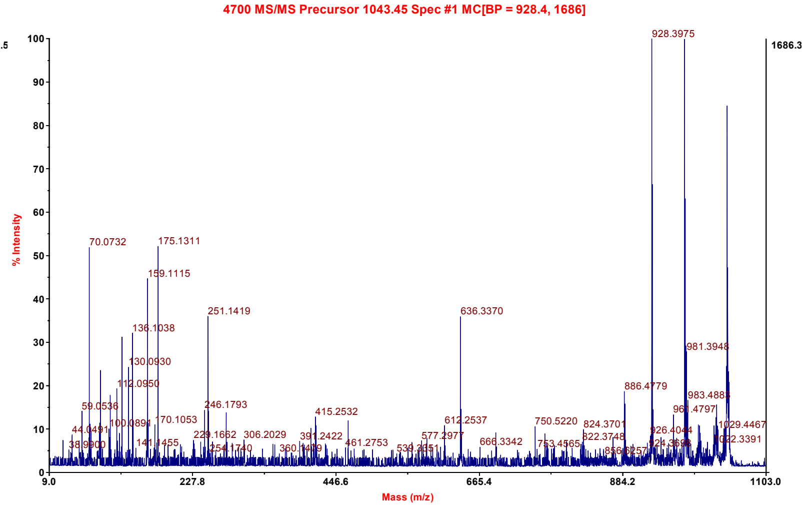

F:\...\J10_MSMS_1043.4532_92.t2d
Acquired:

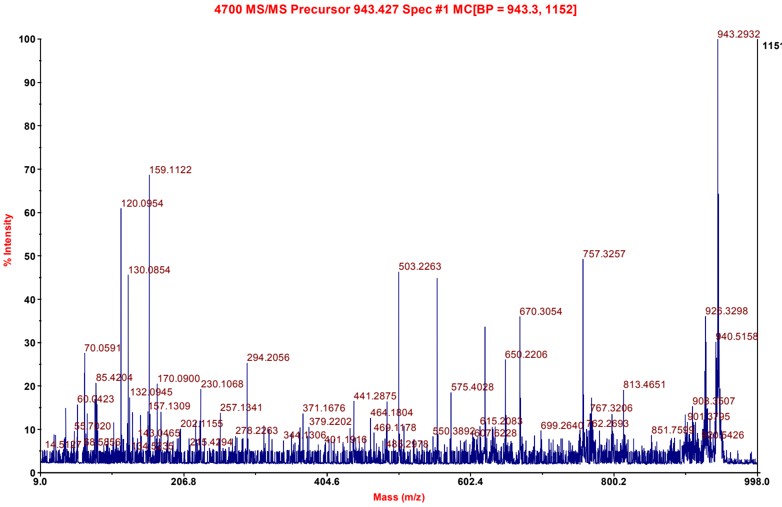

F:\...\J10_MSMS_943.4269_88.t2d
Acquired:

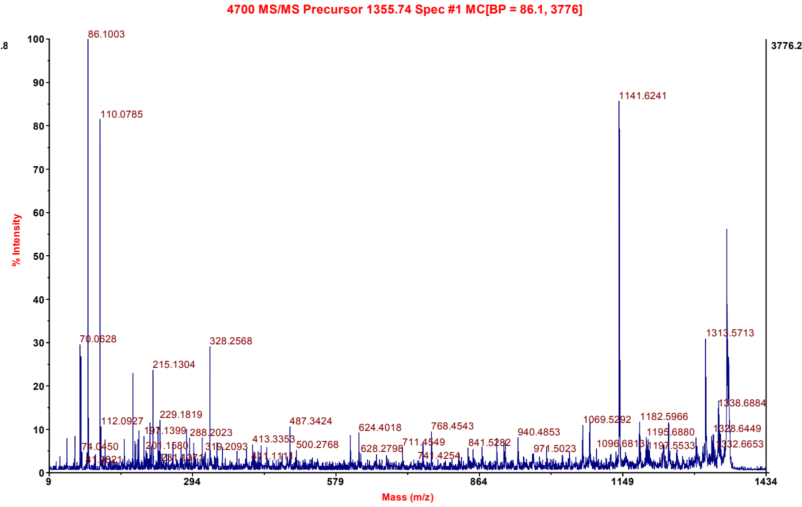

F:\...\J10_MSMS_1355.7419_107.t2d
Acquired:

Figure S19

4700 MS/MS Precursor 1850.94 Spec #1 MC[BP = 1735.8, 545]

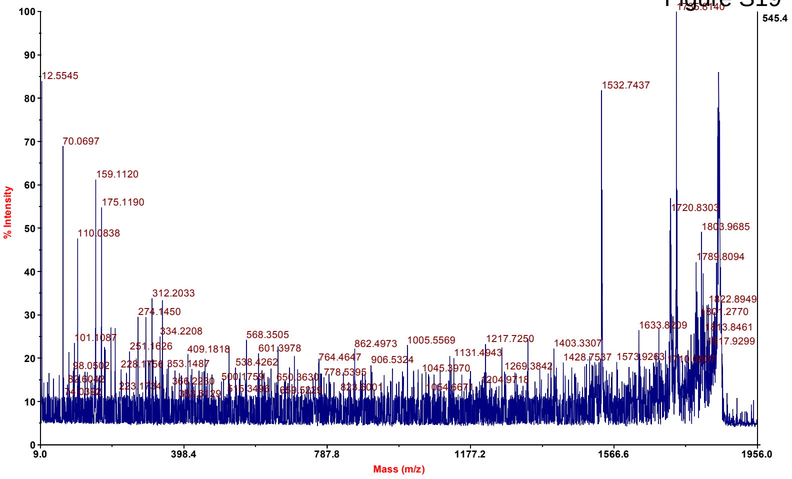

F:\...\J10_MSMS_1850.9360_94.t2d
Acquired:

4700 MS/MS Precursor 1825.8 Spec #1 MC[BP = 1195.6, 892]

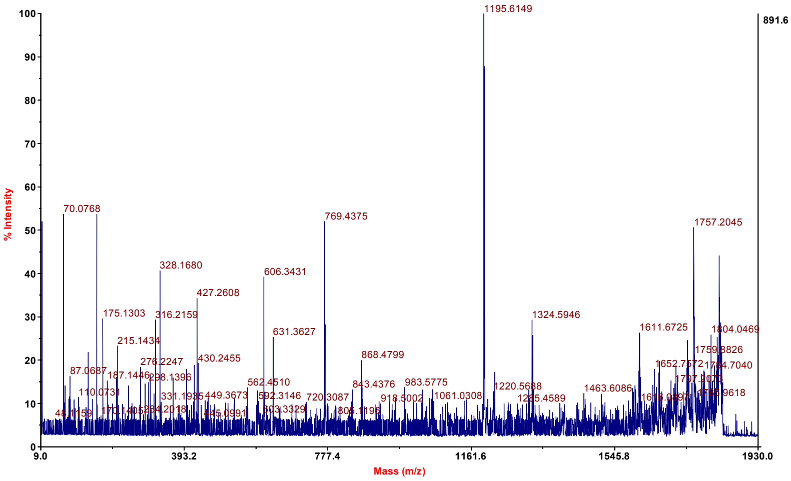

F:\...\J10_MSMS_1825.8009_101.t2d
Acquired:

4700 MS/MS Precursor 1803.89 Spec #1 MC[BP = 1648.8, 1479]

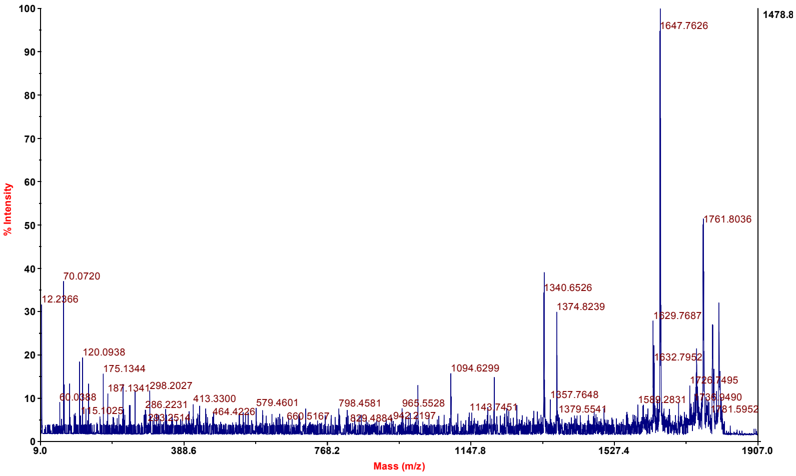

F:\...\J10_MSMS_1803.8860_103.t2d
Acquired:

4700 MS/MS Precursor 1647.75 Spec #1 MC[BP = 464.3, 2224]

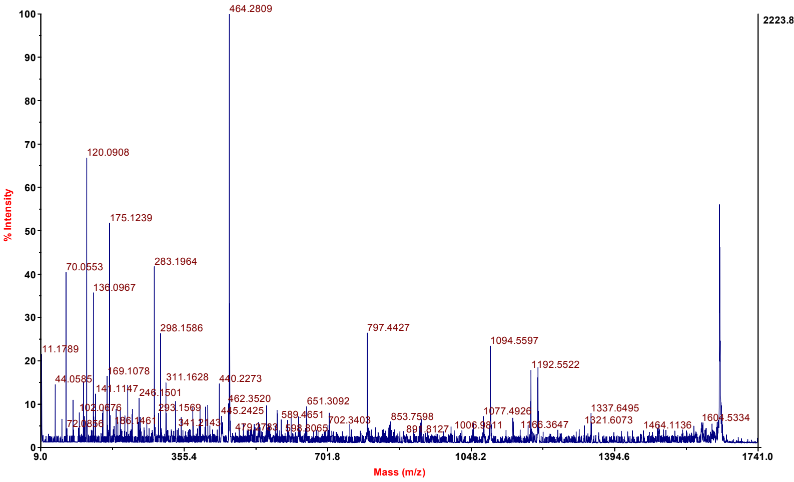

F:\...\J10_MSMS_1647.7499_111.t2d
Acquired:

4700 MS/MS Precursor 1560.81 Spec #1 MC[BP = 1374.7, 3259]

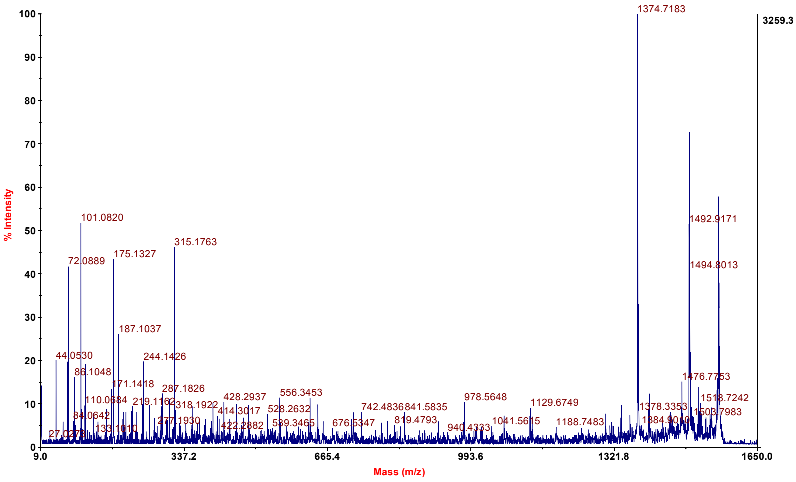

F:\...\J10_MSMS_1560.8083_99.t2d
Acquired:

4700 MS/MS Precursor 1452.71 Spec #1 MC[BP = 110.1, 1030]

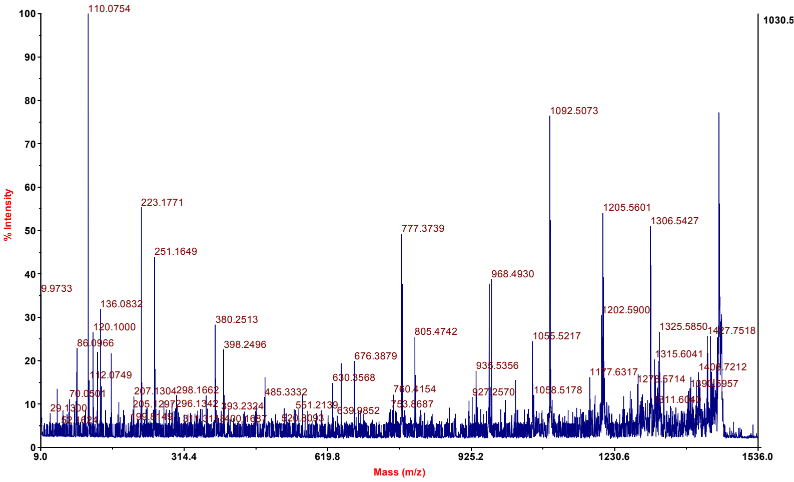

F:\...\J10_MSMS_1452.7109_96.t2d
Acquired:

4700 MS/MS Precursor 1427.73 Spec #1 MC[BP = 1299.5, 4365]

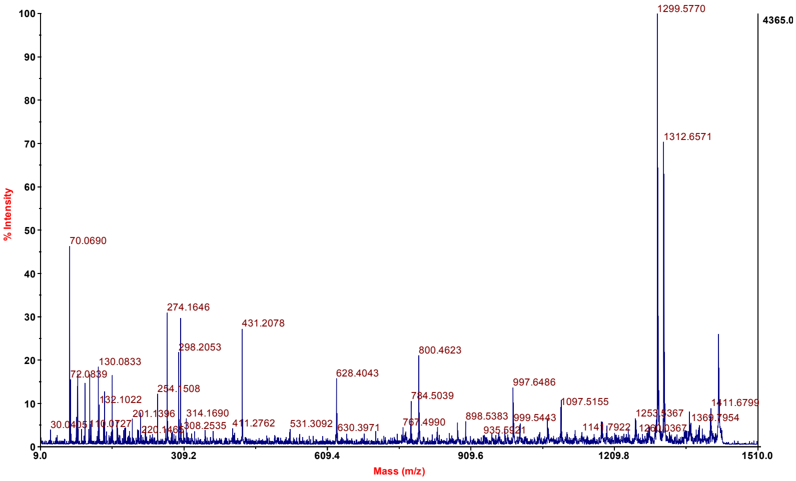

F:\...\J10_MSMS_1427.7281_106.t2d
Acquired:

4700 MS/MS Precursor 1882.93 Spec #1 MC[BP = 12.8, 463]

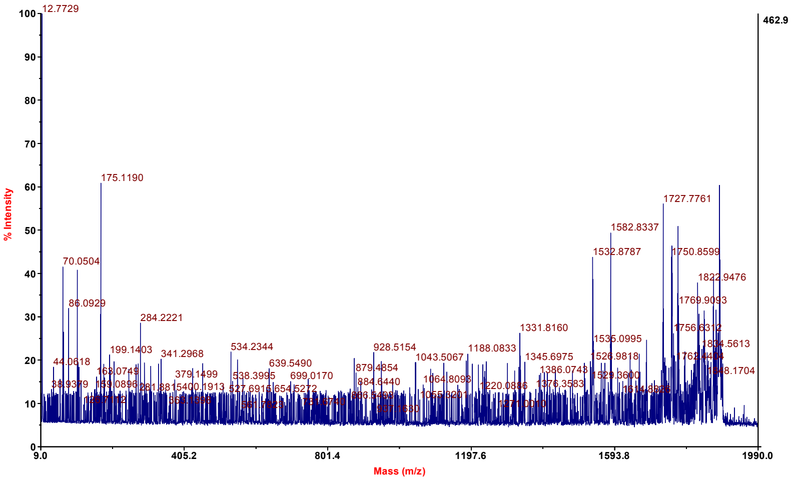

F:\...\J10_MSMS_1882.9298_104.t2d
Acquired:
